# A complementary study approach unravels novel players in the pathoetiology of Hirschsprung disease

**DOI:** 10.1371/journal.pgen.1009106

**Published:** 2020-11-05

**Authors:** Tanja Mederer, Stefanie Schmitteckert, Julia Volz, Cristina Martínez, Ralph Röth, Thomas Thumberger, Volker Eckstein, Jutta Scheuerer, Cornelia Thöni, Felix Lasitschka, Leonie Carstensen, Patrick Günther, Stefan Holland-Cunz, Robert Hofstra, Erwin Brosens, Jill A. Rosenfeld, Christian P. Schaaf, Duco Schriemer, Isabella Ceccherini, Marta Rusmini, Joseph Tilghman, Berta Luzón-Toro, Ana Torroglosa, Salud Borrego, Clara Sze-man Tang, Mercè Garcia-Barceló, Paul Tam, Nagarajan Paramasivam, Melanie Bewerunge-Hudler, Carolina De La Torre, Norbert Gretz, Gudrun A. Rappold, Philipp Romero, Beate Niesler

**Affiliations:** 1 Department of Human Molecular Genetics, Heidelberg University Hospital, Heidelberg, Germany; 2 Lleida Institute for Biomedical Research Dr. Pifarré Foundation (IRBLleida), Lleida, Spain; 3 nCounter Core Facility, Department of Human Molecular Genetics, Heidelberg University Hospital, Heidelberg, Germany; 4 Centre for Organismal Studies, Heidelberg University, Heidelberg, Germany; 5 FACS Core Facility, Campus Heidelberg, Germany; 6 Institute of Pathology, Heidelberg University Hospital, Heidelberg, Germany; 7 Pediatric Surgery Division, Heidelberg University Hospital, Heidelberg, Germany; 8 Pediatric Surgery, University Children's Hospital, Basel, Switzerland; 9 Department of Clinical Genetics, Erasmus University Medical Center, Rotterdam, The Netherlands; 10 Department of Molecular and Human Genetics, Baylor College of Medicine, Houston, Texas, United States of America; 11 Baylor Genetics Laboratories, Houston, Texas, United States of America; 12 Institute of Human Genetics, Heidelberg University Hospital, Heidelberg, Germany; 13 Department of Neuroscience, University Medical Center, Groningen, The Netherlands; 14 UOSD Genetica e Genomica delle Malattie Rare, IRCCS, Instituto Giannina Gaslini, Genova, Italy; 15 Center for Human Genetics and Genomics, New York University School of Medicine, United States of America; 16 Department of Maternofetal Medicine, Genetics and Reproduction, Institute of Biomedicine of Seville (IBIS), University Hospital Virgen del Rocío/CSIC/University of Seville, Seville, Spain; 17 Centre for Biomedical Network Research on Rare Diseases (CIBERER), Seville, Spain; 18 Department of Surgery, Li Ka Shing Faculty of Medicine, The University of Hong Kong, Hong Kong, China; 19 Division of Theoretical Bioinformatics, German Cancer Research Center, Heidelberg, Germany; 20 Genomics and Proteomic Core Facility, German Cancer Research Center, Heidelberg, Germany; 21 Center of Medical Research, Medical Faculty Mannheim, Mannheim, Germany; 22 Interdisciplinary Center for Neurosciences, University of Heidelberg, Heidelberg, Germany; Johns Hopkins University, UNITED STATES

## Abstract

Hirschsprung disease (HSCR, OMIM 142623) involves congenital intestinal obstruction caused by dysfunction of neural crest cells and their progeny during enteric nervous system (ENS) development. HSCR is a multifactorial disorder; pathogenetic variants accounting for disease phenotype are identified only in a minority of cases, and the identification of novel disease-relevant genes remains challenging. In order to identify and to validate a potential disease-causing relevance of novel HSCR candidate genes, we established a complementary study approach, combining whole exome sequencing (WES) with transcriptome analysis of murine embryonic ENS-related tissues, literature and database searches, *in silico* network analyses, and functional readouts using candidate gene-specific genome-edited cell clones. WES datasets of two patients with HSCR and their non-affected parents were analysed, and four novel HSCR candidate genes could be identified: *ATP7A*, *SREBF1*, *ABCD1* and *PIAS2*. Further rare variants in these genes were identified in additional HSCR patients, suggesting disease relevance. Transcriptomics revealed that these genes are expressed in embryonic and fetal gastrointestinal tissues. Knockout of these genes in neuronal cells demonstrated impaired cell differentiation, proliferation and/or survival. Our approach identified and validated candidate HSCR genes and provided further insight into the underlying pathomechanisms of HSCR.

## Author summary

Hirschsprung disease (HSCR) is a rare developmental disorder. It leads to the absence of enteric nerve cells (aganglionosis) in the large intestine and is caused by functional defects of neuronal precursor cells during embryonic development of the gut nervous system. The aganglionosis manifests as a variety of symptoms including impaired peristalsis and the formation of a pathogenic dilatation of the intestine (megacolon). The etiology of HSCR is considered to be multifactorial. Variants in more than 20 genes have been reported to be overrepresented in HSCR and replicated in independent cohorts. However, variants in those risk genes account for only 30% of all cases, suggesting that many more genes have to be implicated in the development of HSCR. As the identification and the subsequent validation of novel gene variants to be disease-causing or not, still remains a major challenge, we established and applied a complementary study pipeline. This enabled us to identify four novel candidate genes in two HSCR patients and to validate their potential disease relevance. Our approach represents a suitable way to dissect the complex genetic architecture underlying HSCR.

## Introduction

The neurocristopathy Hirschsprung disease (HSCR, OMIM 142623), also termed congenital intestinal aganglionosis, represents one of the main causes for neonatal intestinal obstruction. It is characterized by the absence of enteric ganglia (aganglionosis) in the distal bowel. Depending on the length of the affected segment, it is categorized as short-segment (S-HSCR; up to 80%), long-segment (L-HSCR; up to 20%), or total colonic aganglionosis (TCA; up to 8%). The diagnosis is often made within the first days after birth when meconium passage is delayed or a megacolon forms. Patients can also suffer from enterocolitis, constipation, abdominal pain, or emesis. So far, the only treatment is to surgically resect the affected aganglionic bowel segment [1–3].

The enteric nervous system (ENS) innervates the gastrointestinal (GI) tract, regulating blood flow, gut motility, peristalsis, ion and fluid homeostasis, and secretion of signaling mediators [4,5]. The ENS originates from vagal neural crest cells (NCCs) during embryonic development, with delamination between E8.5 and E9.5 in mice and before week 4 of gestation in humans [3,4]. NCC-derived progenitor cells that invade and colonize the developing gut are enteric neural crest-derived cells (ENCDCs). They give rise to enteric neurons and glia cells, which are organized into the submucosal and myenteric plexus ganglia [4,6,7]. Impaired migration, proliferation, survival, or differentiation of NCCs or ENCDCs in the developing lower GI tract may cause the enteric neuropathy HSCR [5,7].

Genetically, HSCR is a rare, multifactorial disorder with an incidence of 1:5,000 live births, showing male sex preponderance, incomplete penetrance, and variable expressivity [1,8,9]. To date, more than 20 genes have been identified and replicated that affect signaling cascades crucial for ENS development. The major susceptibility locus is *RET* as mutations account for up to 50% of familial and 20% of sporadic HSCR cases. To date, mutations in other HSCR risk genes have been identified in about 5% of patients [9,10]. However, variants in these established genes account for only 30% of patients, but many more genes have been implicated in its pathoetiology [9,11]. Identifying disease-relevant variants remains a major challenge.

In the present study, we used an evidence-based procedure to select HSCR candidate genes identified by whole exome sequencing (WES). To prioritize and validate candidate genes, we evaluated bioinformatically filtered WES data based on literature and database searches, and on expression analyses of embryonic ENS tissues. To accumulate further evidence for the potential disease-relevance of our selected candidate genes, WES datasets of further HSCR patients as well as of individuals presenting mostly with a neurological phenotype were taken into account, allowing the identification of further rare variants. Candidate genes were functionally analyzed using gene-specific CRISPR/Cas9-based *knockout* (*KO*) cell clones. We focused on properties relevant to HSCR pathoetiology, such as migration, proliferation, cell survival, and differentiation.

Our approach effectively and comprehensively evaluated novel candidate HSCR genes and may serve as a blueprint for prospective studies aiming to discover and validate novel disease-causing HSCR genes.

## Results

### Candidate gene identification and selection

**Fig 1:**
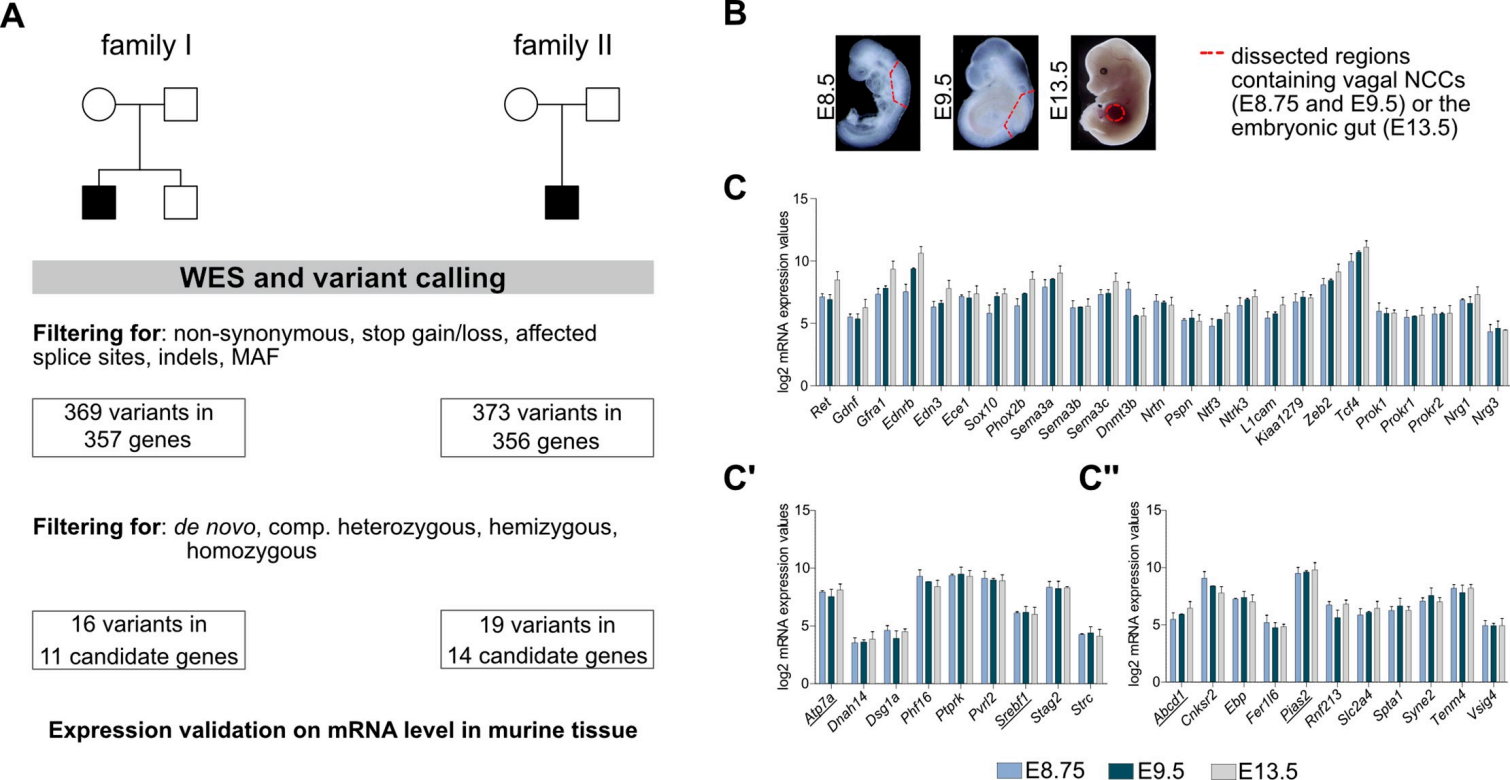
WES data and microarray analysis. (A) Whole exome sequencing (WES) of two sporadic L-HSCR cases in a trio design was followed by variant calling to identify putative HSCR candidate genes. After filtering for respective mutation types, 11 candidate genes remained for patient I and 14 for patient II. (B) For expression validation of filtered candidate genes, transcriptional profiles of relevant embryonic murine tissues were assessed by microarray analysis: vagal NCCs at E8.75/E9.5, embryonic gut at E13.5. (C, C', C") Microarray analyses of embryonic murine tissue showing mRNA expression values of known HSCR risk genes (C), and candidate gene homologs of patient I (C') and patient II (C") (selected candidates for follow-up analyses are underscored) (C, C', C": n = 3, mean + standard error of mean (SEM)). Fig 1B modified from: https://www.hhmi.org/content/zhang-yi-research-abstract-slideshow. MAF: minor allele frequency, NCC: neural crest cell.

Coding variants of two sporadic L-HSCR patients and their non-affected parents were assessed by trio WES. Bioinformatic filtering identified 369 potential disease-causing, rare, coding, non-synonymous SNVs and indels in 357 genes for patient I and 373 in 356 genes for patient II (Fig 1A, S1 Fig). Obtained variants were filtered based on the disease model (including *de novo* for autosomal dominant, homozygous or compound heterozygous for autosomal recessive, and hemizygous for X-linked), resulting in 16 candidate variants in 11 genes for patient I and 19 candidate variants in 14 genes for patient II (Fig 1A, S1 Table, S2 Table, S1 Fig). Of note, none of the filtered candidates were known HSCR genes. However, targeted screening for ENS-relevant and HSCR risk genes (S3 Table) revealed three heterozygous variants for patient I (*ECE1* (rs141146885), *SERPINI1* (rs61750375), and *SUFU* (rs34135067)) and one heterozygous variant for patient II (*EDNRB* (rs780841273)).

To validate this *in silico* selection and to investigate, whether remaining candidate genes are expressed in disease-relevant stages and tissues, transcriptional profiles of three murine embryonic ENS-relevant tissues (E8.75 and E9.5 vagal NCCs, and E13.5 gut) were assessed (Fig 1B). Besides the expression of murine gene orthologues of already confirmed HSCR genes (*Ret*, *Gdnf*, *Gfra1*, *Ednrb*, *Edn3*, *Ece1*, *Sox10*, *Phox2b*, *Sema3a*, *Sema3b*, *Sema3c*, *Dnmt3b*, *Nrtn*, *Pspn*, *Ntf3*, *Nrk3*, *L1cam*, *Kiaa1279*, *Zeb2*, *Tcf4*, *Prok1*, *Prokr1*, *Prokr2*, *Nrg1*, *Nrg3*) [10] (Fig 1C), comparable expression levels could also be detected for murine gene orthologues of our selected candidate genes (9/11 for patient I, 11/14 for patient II) (Fig 1C' and 1C"). The obtained results verified our selection, as orthologues of candidates were all expressed at embryonic stages and tissues relevant for disease manifestation (S1 Fig).

### Prioritization of novel HSCR candidate genes

Literature and database searches were performed to prioritize candidate genes. Further, genes presenting with variants of CADD scores above 13 were taken into account, which indicate that the variant is among the 5% most deleterious substitutions in the human genome [12]. 14 out of 16 variants of patient I as well as 16 out of 19 variants of patient II reached this CADD score threshold (S1 Table, S2 Table). Moreover, we considered central nervous system (CNS) phenotypes, finally leading to 4 candidates in patient I and 9 candidates in patient II (S1 Table, S2 Table, S1 Fig).

In the filtered list of candidate genes, two genes per patient were prioritized for detailed characterization, and variants were confirmed by Sanger sequencing: ***ATP7A*** (*ATPase COPPER TRANSPORTING ALPHA*) and ***SREBF1*** (*STEROL REGULATORY ELEMENT-BINDING PROTEIN 1*) for patient I, and ***ABCD1*** (*ATP-BINDING CASSETTE SUBFAMILY D MEMBER 1*) and ***PIAS2*** (*PROTEIN INHIBITOR OF ACTIVATED STAT 2*) for patient II (S1 Table, S2 Table, S2 Fig).

### Genetic evaluation

**Fig 2:**
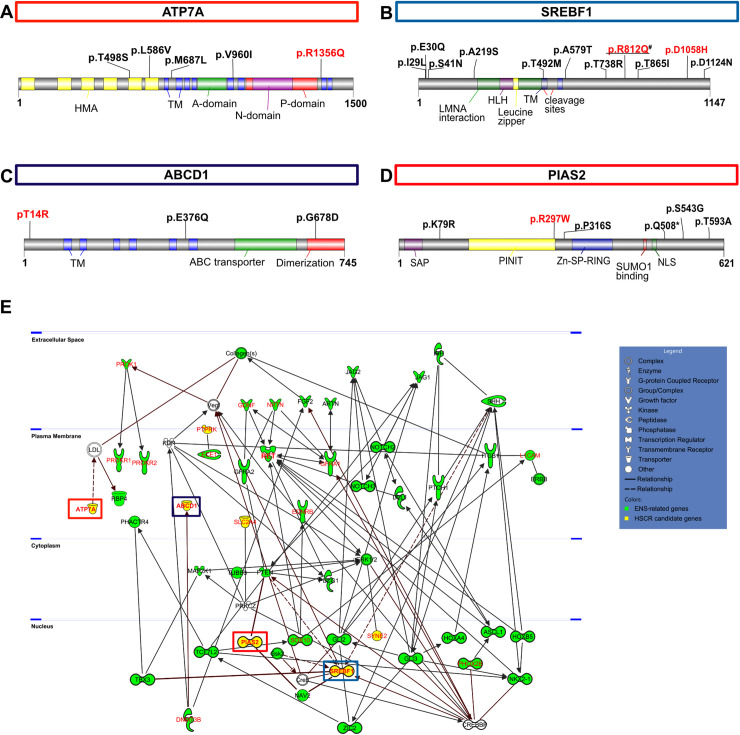
Genetic evaluation of candidate genes and network analyses. (A-D) Disease relevance of all candidate genes was examined by evaluating genetic data from additional patients with HSCR. Variants shown in red were detected in index cases of this study while filtered variants from other cohorts are shown in black (# variant was found in both). More information about the filtered rare variants is given in S4 Table. Protein domain structures and the localization of filtered rare variants are given for ATP7A (A), SREBF1b (B), ABCD1 (C), and PIAS2ß (D). Functional domains in specific isoforms were annotated based on database entries (Pfam, NCBI, Uniprot) or additional literature findings [53,54]. HMA: heavy metal associated, TM: transmembrane, A: actuator, N: nucleotide, P: phosphorylation, HLH: helix loop helix, LMNA: LAMIN A/C, ABC: ATP-binding cassette, SAP: scaffold attachment factor-A/B, acinus and PIAS, PINIT: Pro-Ile-Asn-Ile-Thr, Zn-SP-RING: zinc binding—Siz/Pias—really interesting new gene, SUMO: small ubiquitin-related modifier, NLS: nuclear localization signal. (E) IPA core analysis was performed using lists of filtered variants detected in both index cases (S1 and S2 Tables), of ENS-relevant and known HSCR risk factors (S3 Table). Indirect interactions were only kept if linked to candidate gene products. ENS-relevant factors are highlighted in green while validated HSCR risk factors are lettered in red. Candidates are colored in yellow, the four selected most promising candidates are marked by red lettering. Protein interactions between candidates and factors of interest are marked in red. Proteins are arranged according to their subcellular localization.

Moreover, screening of WES datasets for all four candidate genes of 767 HSCR patients revealed additional rare variants, although the variants present in patients I and II were not seen. Based on our filtering criteria, 6 non-synonymous variants for *ATP7A*, 10 for *SREBF1*, 2 and 5 for *ABCD1* and *PIAS2* respectively could be found (Fig 2A–2D, S4 Table, S1 Fig).

### Candidate gene validation

#### IPA network analysis

To better understand the biological context of selected candidates, we performed a network analysis using the IPA software tool. All four candidates showed multiple direct and indirect protein interactions with ENS-relevant and HSCR risk factors (Fig 2E). ATP7A is indirectly connected to RBP4 via the LDL complex. The very long chain fatty acid (VLCFA) transporter ABCD1 is directly connected to the HSCR factor DNMT3B, while several direct connections for SREBF1 and PIAS2 were found. Among others, both candidates are directly connected to the HSCR-associated factor NAV2. Additionally, PIAS2 was identified as a SUMOylation mediator of PTEN (Fig 2E, S1 Fig).

#### Screening of additional WES and WGS data

To investigate, whether our selected candidates might be involved in ENS- and CNS-related phenotypes alike, we subsequently applied the same search and filtering strategy to a set of approximately 15,500 cases submitted for clinical WES and WGS (S1 Fig). This cohort is clinically diverse, with a majority of cases being submitted for neurological indications. In total, we identified 114 individuals harboring coding non-synonymous or indel variants in one of our four selected candidate genes. Of these, 37 individuals carried variants in *ATP7A*, 31 in *SREBF1*, 33 in *ABCD1* and 13 in *PIAS2*. Investigation of the clinical indications listed by the referring provider showed that 101 out of 114 cases presented with a GI phenotype (*ATP7A*: 30; *SREBF1*: 30; *ABCD1*: 28; *PIAS2*: 13). Furthermore, 11 individuals had autism spectrum disorder (ASD) listed as a clinical indication (*ATP7A*: 4; *SREBF1*: 3; *ABCD1*: 2; *PIAS2*: 2), and 28 individuals were diagnosed with intellectual disability (*ATP7A*: 9; *SREBF1*: 3; *ABCD1*: 14; *PIAS2*: 2) (S5 Table).

#### GTEx database analysis

In line with this, we also considered expression data in the GTEx database (human brain, colon tissue) to verify their relevance in the brain and the gut and could detect a positive expression in both tissues (S7 Table, S1 Fig).

#### Expression analyses in murine and human tissues

Immunofluorescence analyses of murine embryos at relevant developmental stages (E9.5, E10.5, E11.5, E13.5) was performed and revealed broad protein expression of all candidates in ENS-related tissue structures and cells (S6 Table, S3A Fig, S3B Fig). Complementary, immunohistochemical analysis also showed candidate gene expression in human fetal colon sections at 25th week of gestation (S3C Fig). Candidate gene expression was also confirmed in human fetal hindgut specimens at 12, 14, and 16 weeks of gestation in published data (S4 Fig) [13].

All validation steps are summarized in S7 Table.

### Determining the neuronal-specific role of candidates

#### Generation of gene-specific *KO* clones

To determine the neuronal functions of selected genes, we generated gene-specific *KO* cell clones of the human neuroblastoma cell line SHSY5Y. The differentiation, migration, proliferation, and survival of these cells were assessed. A *KO* clone of the major HSCR gene *RET* was analyzed as a proof-of-principle control, and all clones were also compared with a *mock control* clone.

*KO* clones either harbored homozygous (*RET KO*, *ABCD1 KO*) or compound heterozygous genome modifications (*ATP7A KO*, *SREBF1 KO*) (S5 Fig). Off-target effects were largely excluded (S1 Data). However, genome editing did not work for *PIAS2* as no *KO* clone with neuronal morphology could be generated, despite several attempts with sgRNAs targeting two different exons. While the genetically modified exon 6 harboring a homozygous one-nucleotide insertion escaped *KO* by alternative splicing, sgRNAs targeting exon 2 did not yield a suitable clone.

#### Neuronal differentiation of *KO* clones

*KO* clones were differentiated into a neuronal-like phenotype to investigate the functional impact of candidate genes on neuronal development (*KO* vs *mock control*).

**Fig 3:**
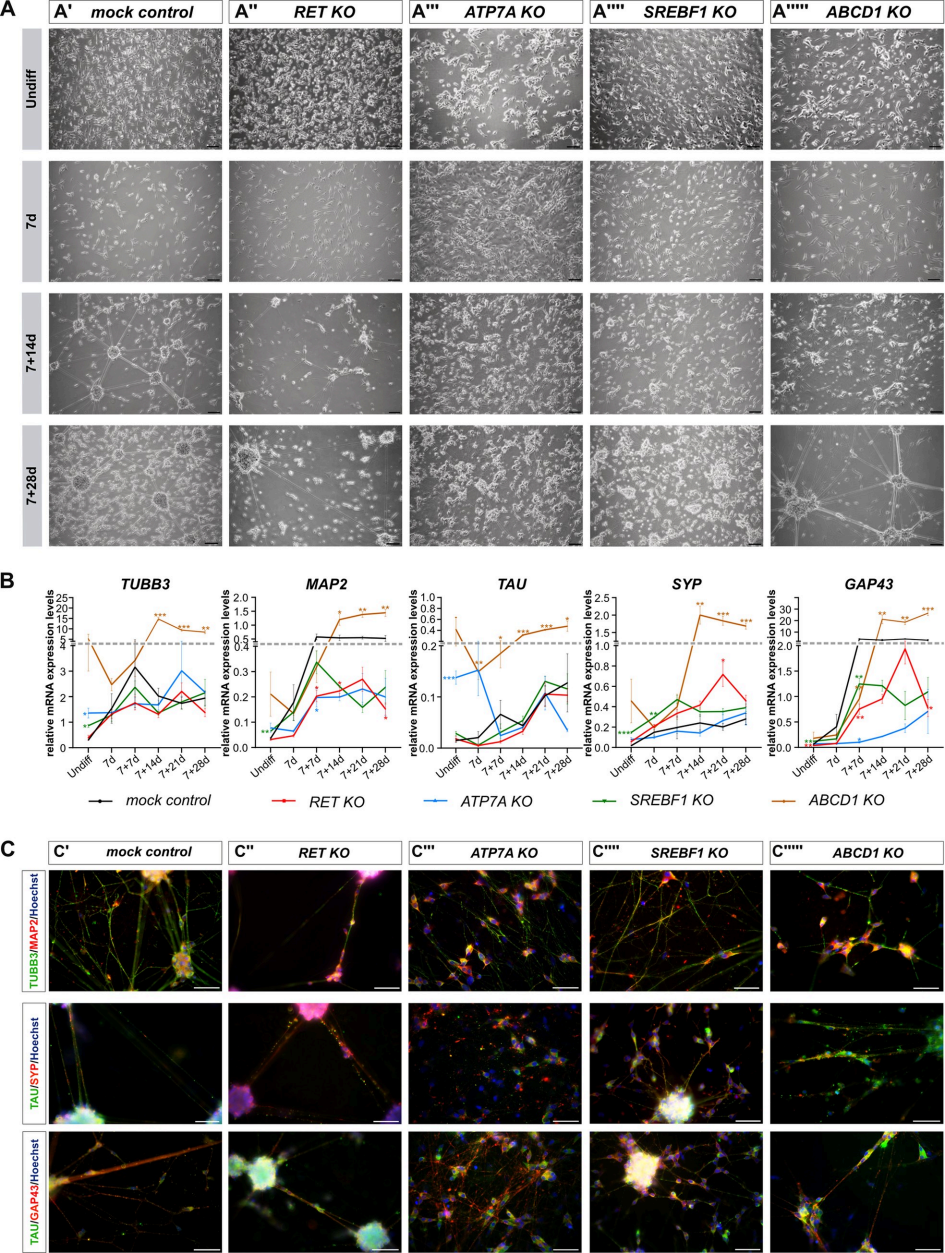
Differentiation of genome-edited clones on morphological and marker expression level. (A) Genome-edited clones and the *mock control* clone were differentiated into a neuronal-like phenotype and cell morphologies were assessed by brightfield microscopy (A': *mock control*, A": *RET KO*, A"': *ATP7A KO*, A"": *SREBF1 KO*, A""': *ABCD1 KO*). Scale bars: 100μm. (B) Gene expression profiles of different neuronal cell fate markers were assessed in differentiating cell clones (*TUBB3*, *MAP2*, *TAU*, *SYP*, *GAP43*) by qRT PCR. (n = 3, mean + standard error of mean (SEM); exploratory data analysis by two-sided unpaired t-Test with/without Welch’s correction, *p<0.05; ** p<0.01; *** p< 0.001). (C) Cells after 7+14d of differentiation were investigated by IF analyses for marker expression patterns. Images are representatives of different experiments. Neuronal-specific markers are indicated in red (MAP2, GAP43, SYP) and green (TUBB3, TAU) (C': *mock control*, C": *RET KO*, C"': *ATP7A KO*, C"": *SREBF1 KO*, C""': *ABCD1 KO*). Nuclei were counterstained with Hoechst 33342 (blue). Scale bars: 50μm. Undiff: undifferentiated.

All four *KO* clones showed impaired development of a neuronal-like phenotype (Fig 3A). The neuronal morphology (elongated cell bodies carrying several neurite-like outgrowths) became apparent from the undifferentiated to the 7d differentiated state in all clones, except for the *ATP7A KO* clone, where a confluent cell monolayer was present after 7d (Fig 3A"'). After 7+14d, only the *mock control* clone had a highly organized network of interconnected neuronal-like clusters (Fig 3A'). *KO* clones for *RET* (Fig 3A"), *SREBF1* (Fig 3A""), and *ABCD1* (Fig 3A""') showed delayed network formation. After 7+28d, all genome-edited clones (Fig 3A), except the *ATP7A KO* clone (Fig 3A"'), showed neuronal-like clusters, which were connected by semi-adherent neurite-like connections.

Throughout differentiation, mRNA expression profiles of various cell type markers were assessed (Fig 3B, S6 Fig). Expression of these markers increased in the *mock control* clone with advancing neuronal-like maturation. While *MAP2*, *TAU*, and *SYP* were only marginally expressed, *TUBB3* and *GAP43* were prominently expressed. Marker expression was significantly different in all gene-specific *KO* clones during neuronal differentiation at various differentiation stages. *MAP2* and *GAP43* expression was significantly reduced at several stages, while *SYP* expression was upregulated after 7+21d in the *RET KO* clone. For the *ATP7A KO* clone, *TUBB3* and *TAU* expression was significantly higher than in the undifferentiated control, but *TAU* expression dropped over time. At 7+7d, *MAP2* and *GAP43* expression was lower. During early differentiation, most markers showed increased expression in the *SREBF1 KO* clone, except for *GAP43* after 7+7d. In contrast, expression of all markers was significantly higher in the *ABCD1 KO* clone during later neuronal development (Fig 3B).

In addition, expression of *P75NTR* and *NES* (neuronal progenitor markers) and *UCHL1* and *ASCL1* (pan-neuronal markers) was investigated in all cell clones (S6 Fig). These analyses are described in detail in the Supplementary (S1 Data).

Complementary immunofluorescence analyses confirmed TUBB3, MAP2, TAU, SYP, and GAP43 marker expression in most cell clones differentiated for 7+14d (Fig 3C). Additionally, cellular morphologies were comparable to previous analyses (Fig 3A). Corresponding to their functions, signals were detectable in all *KO* clones in neurite-like projections (TUBB3, MAP2, GAP43 and TAU), while for the synapse-marker SYP, a dotted-like staining pattern was observed along these structures (Fig 3C). However, for the *ATP7A KO* clone, no neuronal-like clusters and no distinct staining pattern for TAU could be detected at this maturation stage (Fig 3C"'). Numerical raw data for Fig 3B and S6 Fig are given in S13 Table.

#### Further comparative functional *in vitro* analyses

**Fig 4:**
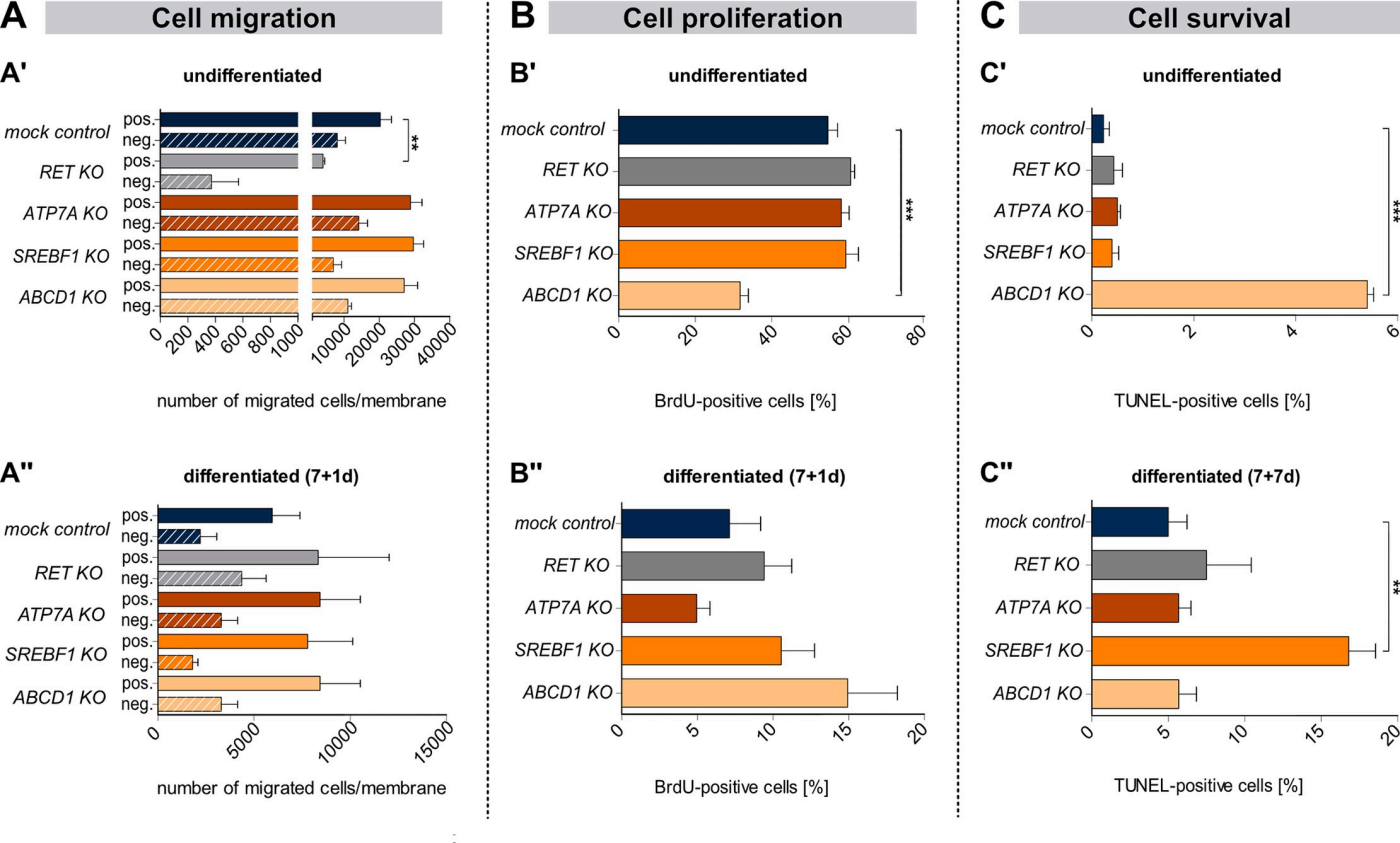
Comparative functional analyses of all genome-engineered clones. (A) Cell migration in undifferentiated (A') and differentiated (7+1d) (A") cell clones was measured by Boyden chamber assays. In the positive control, FBS was applied as a chemoattractant for undifferentiated cells and BDNF was applied as a chemoattractant for differentiated cells (negative: basal medium only). (B) The proliferation capacity of undifferentiated (B') and differentiated (7+1d) (B") genome-edited clones was assessed by a BrdU assay. (C) The number of apoptotic cells in undifferentiated (C') and differentiated (7+7d) (C") clones was determined by a TUNEL assay. (A-C) All functional analyses were compared with the *mock control* clone. Bar plots show mean + standard error of mean (SEM) (n≥3). One-way ANOVA, Bonferroni corrected, *p<0.05; **p<0.01; ***p< 0.001. Undiff: undifferentiated.

Cell migration (Fig 4A), proliferation (Fig 4B), and survival (Fig 4C) were compared in undifferentiated and differentiated *KO* clones and the *mock control* clone. Cell migration was significantly reduced in the undifferentiated *RET KO* clone (Fig 4A'), but no difference was observed in differentiated clones (Fig 4A"). In addition, significantly fewer proliferative cells (BrdU^+^) were detected in the undifferentiated *ABCD1 KO* clone (Fig 4B'), whereas no difference was observed in differentiated (7+1d) candidate clones (Fig 4B"). Apoptosis was higher in the undifferentiated *ABCD1 KO* clone (Fig 4C'), and the differentiated (7+7d) *SREBF1 KO* clone (Fig 4C"). Respective numerical data are summarized in S13 Table.

## Discussion

Identifying and validating disease-relevant genetic variants in complex disorders such as HSCR remains challenging. To overcome these obstacles, we established a complementary approach to select candidate genes, to collect gene-related information on multiple levels, and finally to determine their disease relevance in functional readouts.

WES data revealed several hundreds of promising HSCR candidate variants in patient I and II. After prioritizing specific mutation types, we further took mRNA expression profiles in murine embryonic tissues, CADD scores (≥13), published literature, and database information into account. We considered extra-intestinal neurological phenotypes to be relevant, since the CNS and ENS are structurally and functionally similar [4,14–16], and interconnections between CNS disorders and HSCR/ENS-relevant phenotypes have already been reported [17,18]. Recently, genes involved in HSCR and ENS development (such as *CHD8* [19] and *NLGN3* [20,21]) were also found to be implicated in neurodevelopmental disorders indicating that genetic neurodevelopmental processes might be conserved between the CNS and ENS. Of note, there is growing evidence suggesting that variants predisposing to ASD by affecting CNS structure and function might also impact ENS development culminating in disturbed GI structure and function [22].

Finally, we ended up with 4 genes for patient I and 9 genes for patient II with potentially relevant variants, that all would equally have qualified for further evaluation. However, since the envisioned functional part of the pipeline was highly demanding, we decided on two candidates per patient: *ATP7A* and *SREBF1* for patient I and *ABCD1* and *PIAS2* for patient II. Having now proven this study pipeline as feasible, it represents a perfect basis for an automated high-throughput approach. Once established, we aim to apply the adapted pipeline to additional candidates in the future.

*ATP7A* encodes a copper transporter which regulates intracellular ion levels and has previously been implicated in Menkes disease [23]. The VLCFA transporter *ABCD1* is involved in X-linked adrenoleukodystrophy (X-ALD), and impaired ABCD1 function causes substrate accumulation and neurodegeneration [24]. *SREBF1* encodes a crucial regulator of sterol and cholesterol biosynthesis, while *PIAS2* encodes an E3-SUMO ligase and has multiple targets besides the STAT family [25,26]. These candidates have also been implicated in neurodegenerative disorders including Alzheimer and Parkinson disease [27,28].

We validated the disease relevance of our selected candidates using multiple approaches. *In silico* network analyses revealed direct and indirect interactions between the candidates and ENS-/HSCR-relevant factors [29–31], suggesting the selected candidates modify ENS-relevant genes, or participate in uncharacterized HSCR susceptibility pathways [1,32]. The disease relevance of our candidates was strengthened by additional variants in other HSCR patients. Importantly, another non-synonymous variant in *ATP7A* was also found in a patient with pseudo-obstruction (rs201788154, c.2452A>G, p.T818A), underlining its potential significance in functional GI disorders.

As already mentioned, neurodevelopmental processes seem to be conserved between the ENS and the CNS. Thus, genetic alterations might affect both systems likewise. In order to get an idea, whether our candidates might be involved in ENS-/CNS-related phenotypes, we screened clinical WES/WGS data of a non-HSCR, neurodevelopmental disease-focused clinical cohort. Obtained results are in line with this, as patients carrying variants in the selected candidates presented not only with neurological phenotypes but also with a high prevalence of GI phenotypes.

We analyzed the protein expression of candidate genes in murine embryonic ENS-relevant tissues and human fetal colon tissue. Although the human specimens did not match the stages examined in murine tissue, expression data from human tissue was valuable because HSCR is a prenatal human disorder. The transcript and protein expression profiles of the four candidates are already known from different species and tissues at various developmental stages [24,33–37]. However, to the best of our knowledge, ENS-specific analyses have not been performed so far.

To correlate candidate gene function with HSCR-causing pathomechanisms, gene-specific *KO* cell clones were generated from SHSY5Y human neuroblastoma cells. Although this CNS-derived cell source does not mimic the ENS, we hypothesized that neuronal differentiation, migration, proliferation, and survival are conserved between the CNS and ENS. This assumption is supported by the previous finding that the same genes are involved in ENS and CNS development. We successfully generated *KO* clones for *ATP7A*, *SREBF1*, *ABCD1* and *RET*, but *PIAS2* escaped all editing approaches. It is tempting to speculate that *PIAS2* has an essential cellular function [38], particularly because the edited neuronal-like clone escaped CRISPR-Cas9-based *KO* by alternative splicing and expressed a shorter, unknown *PIAS2* isoform.

To assess phenotypic alterations resembling HSCR pathogenesis *in vitro*, we also studied neuronal differentiation, migration, proliferation, and survival in *KO* clones compared with a *mock control* and *RET KO* clone. Most striking phenotypic alterations for the *RET KO* clone were shown for its neuronal differentiation and migration capacity. These findings were in agreement with two previous studies–one using induced pluripotent stem (iPS) cell-derived ENCDCs from a HSCR patient with a *RET* mutation and one using neuronal precursors from *Ret*-deficient mice [39,40]. In addition, neuronal markers were differentially expressed in the *RET KO* clone, underlining the suitability of our *in vitro* approach in assessing neuronal-specific impairments. We did not validate the neuronal markers on the protein level, but the mRNA expression profiles (e.g. *GAP43*) were consistent with results from previous studies [41]. How *RET* deficiency influences marker expression in the differentiating *KO* clone and the functional relevance of this dysregulation should be elucidated in future studies.

Candidate gene-specific *KO* clones displayed altered cellular functions. The most striking morphological difference was seen in the *ATP7A KO* clone, which displayed high cell densities in line with neuronal-like maturation. Although we did not detect changes in proliferation, the higher cell densities may have been caused by the inverse relationship between ATP7A expression and proliferation in neuroblastoma cells [33]. *In vivo* analyses using a murine disease model demonstrated that Atp7a might be involved in axonal outgrowth and synaptogenesis [42]. This may explain the significantly reduced *MAP2* and *GAP43* expression in differentiating cells.

The *SREBF1 KO* clone showed altered differentiation and survival compared with the *mock control* clone. Srebf1 is involved in lipogenesis, so may be crucial for dendritogenesis [43]. Others have shown that *Srebf1c KO* causes peripheral neuropathy in mice [44] and that *SREBF1* is crucial for cancer cell growth and viability [45,46]. The observed defects in our *SREBF1 KO* clone are thus in line with its known protein functions.

Further, *ABCD1* deficiency reduced proliferation and increased apoptosis in undifferentiated cells. In contrast, neuronal-like network formation was only slightly altered and mRNA expression of many neuronal markers was mostly upregulated during differentiation. These findings may be explained by a compensatory effect of *ABCD2* in the *KO* clone which might be launched during neuronal cell fate induction [47,48]. In the X-ALD zebrafish *abcd1 KO* model, oligodendrocytes showed increased apoptotic rates [36]. We observed a comparable defect in our neuronal-like *ABCD1 KO* clone.

Along with the difficulty pinpointing HSCR-relevant candidate genes, the complex pathogenesis of HSCR is multifactorial [8,10]. To get a complete pathophysiological picture in future studies, rare coding variants (SNVs) cannot be studied alone; structural alterations (CNVs), common non-coding risk alleles, and rare coding variants affecting ENS-relevant genes should also be considered. To further strengthen the importance of our four selected candidate genes in HSCR, transcriptome analyses and protein analyses in genome-edited cell clones must be performed. In addition, genome-edited primary ENS-derived cells or patient-derived iPS cells could reveal how the four genes contribute to underlying disease-causing pathomechanisms. Finally, rescue experiments using isogenic iPS cell controls could functionally validate selected candidate genes.

**Fig 5:**
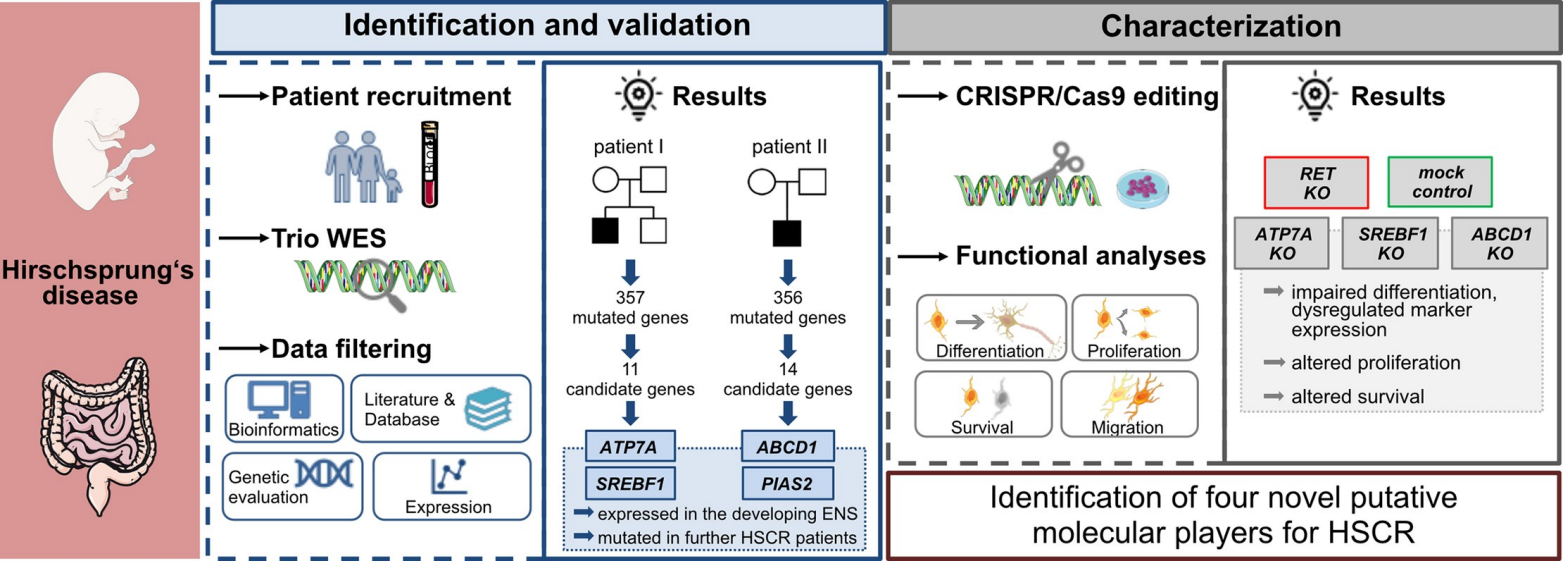
Study summary. HSCR candidate gene identification and validation was achieved by trio WES and various data filtering steps. Two promising candidate genes were selected per patient (*ATP7A*, *SREBF1* for patient I; *ABCD1*, *PIAS2* for patient II) and were characterized using gene-specific *KO* cell clones. Functional analyses revealed impaired neuronal functions thereby validating the selected genes as putative molecular players for HSCR. Figure components were kindly provided from Microsoft Office Power Point and Servier Medical Art (https://smart.servier.com/). This work is licensed under the Creative Commons Attribution 3.0 Unported License. To view a copy of this license, visit http://creativecommons.org/licenses/by/3.0/ or send a letter to Creative Commons, PO Box 1866, Mountain View, CA 94042, USA. The authors acknowledge the free figure access.

Taken together, we have identified four novel HSCR risk genes based on two patients with L-HSCR by evaluating trio WES data and focusing on genes that harbor rare SNVs. We corroborated the potential disease-relevance of three selected genes using various *in vitro* analyses (Fig 5) and suggest that each of the identified variants might contribute to disease risk based on the multifactorial origin of HSCR. Our approach represents a suitable tool identifying candidate genes and evaluating their disease relevance in enteric neuropathies such as HSCR. We plan to adapt our approach to larger studies of candidate genes by up-scaling and automating certain steps. In this way, we hope to understand the complex genetic architecture underlying HSCR and to facilitate the development of novel therapies.

## Materials and methods

### Ethics statement

The parents provided written informed consent for genetic and molecular analyses, including WES. The study was approved by the Ethical Board of the Medical Faculty of the University Hospital Heidelberg (S509/2012).

### Candidate gene identification and selection

#### HSCR patients

Both HSCR patients were full-term neonates with a birthweight of 3,350 g (patient I) and 3,470 g (patient II), and presented with long-segment aganglionosis (L-HSCR) at least up to the colon transversum. In patient I, laparoscopic-assisted transanal endorectal pull-through (TERPT) with simultaneous colostomy closure was performed at 14 weeks of age at the Pediatric Surgery Clinic (University Hospital Heidelberg, Germany). Patient II underwent an assisted pull-through Swenson procedure 8 months after diagnosis in another clinic. After colostomy closure, the child suffered persistently from intestinal obstruction with severe constipation. TERPT was repeated in Heidelberg at one year of age after persistent aganglionosis was diagnosed.

#### WES and variant calling

In a trio design, patients with HSCR and their non-affected parents were analyzed by WES. The Agilent SureSelect V4 kit (Agilent Technologies, Santa Clara, USA) was used to prepare the sequencing library. Sequencing was performed on the Illumina HiSeq 2500 platform (Illumina, San Diego, USA) as described in the Supplementary (S1 Text). Filtered variants for both patients (patient I: 11 genes, patient II: 14 genes) are shown in S1 Table and S2 Table.

#### Transcriptomics analysis in murine tissues

For microarray profiling, embryonic regions of interest (pre-migratory vagal NCCs at stages E8.75 and E9.5; embryonic gut at E13.5, without stomach) were prepared and collected in 1 mL of TRIzol reagent (Thermo Fisher Scientific, Waltham, USA). Embryonic tissues from each litter were pooled and three litters were analyzed per stage. Total RNA was extracted according to the manufacturer’s instructions. For transcriptomics analyses, 500 ng of total RNA was used for evaluation with the mouse Clariom S array (Thermo Fisher Scientific) as described in the Supplementary (S1 Text).

### Prioritization of novel HSCR candidate genes

CADD scores of single nucleotide variants were calculated using the CADD model GRCh37-v1.4 (https://cadd.gs.washington.edu/snv), while CADD version 1.3 was installed locally for indels (S1 Table, S2 Table, S1 Fig).

The relevance of filtered candidate genes in neurological diseases was assessed based on published literature and the Genetic Association and the Disease Gene databases (https://geneticassociationdb.nih.gov/; https://www.disgenet.org/).

### Genetic evaluation of candidate genes

Available WES and whole genome sequencing (WGS) data of 767 HSCR patients [8,49] from groups of the International HSCR Consortium (Stanislas Lyonnet, http://www.erare.eu/ financed-projects/hscr) were analyzed for the presence of rare exonic variants in the candidate genes (*ATP7A*, *SREBF1*, *ABCD1*, *PIAS2*). Common variants/SNPs (MAF >1%) were already excluded in individual studies. First, all variants, except for non-synonymous (missense mutations, stop loss and stop gain), were removed. Next, remaining variants were compared with variant data deposited in the gnomAD browser (population-matched comparison). Variants were kept, if the MAF was < 1%. To assess the putative functional relevance of the filtered variants, CADD scores were calculated using the CADD model GRCh37-v1.4 (https://cadd.gs.washington.edu/snv).

### Candidate gene validation

#### IPA network analysis

To determine the biological context of selected candidate genes, a network analysis was performed. A combined list of filtered WES datasets (n = 25, final candidate genes from both patients), ENS-relevant genes (n = 117), and HSCR-relevant genes (n = 25) [10,16,50] was uploaded to the Ingenuity Pathway Analysis (IPA) software (Qiagen, Venlo, The Netherlands) (S3 Table) and analyzed (S1 Text).

#### Screening of additional WES and WGS data

To assess the potential involvement of selected candidate genes in ENS- and CNS-related phenotypes, clinical WES and WGS data of approximately 15.500 cases at Baylor Genetics Laboratories (Houston, TX, USA) were screened according to the previously applied filtering criteria. WES had been performed corresponding to previously described methods [51].

#### GTEx database analysis

Furthermore, publicly available human brain and colon mRNA expression profiles (GTEx database, https://www.gtexportal.org/home/) of the four selected candidates were considered.

#### Protein expression analyses in murine tissues

For comparative immunofluorescence analyses, whole murine embryos (E9.5, E10.5, E11.5, E13.5) were prepared and examined as described in the Supplementary (S1 Text, S9 Table, S10 Table).

#### Protein expression analyses in human tissues

For expression analyses in human specimens, formalin-fixed paraffin-embedded (FFPE) colon tissue of a fetus acardius amorphus (25^th^ week of gestation) was used [52]. FFPE sections were stained by immunohistochemistry (S1 Text, S9 Table, S10 Table). A summary of the complete study approach is illustrated schematically in S1 Fig.

### Candidate gene characterization

Candidate genes were characterized using SHSY5Y cells (cultivated and processed as indicated in the Supplementary (S1 Text, S8 Table, S11 Table, S12 Table)), including CRISPR/Cas9 genome editing and functional analyses.

#### Expression analyses of genome-edited clones

For mRNA expression analyses of SHSY5Y clones, cells were harvested and collected in TRIzol. Total RNA was extracted using a modified version of the RNAqueous-Micro Total RNA Isolation Kit protocol (Thermo Fisher Scientific). Protein and mRNA expression analyses are described in the Supplementary (S1 Text, S8 Table, S9 Table, S10 Table).

#### Functional *in vitro* assays

Migration of undifferentiated and differentiated (7+1d) SHSY5Y cells was investigated using Boyden chamber assays as outlined in the Supplementary (S1 Text).

Proliferation of undifferentiated and differentiated (7+1d) SHSY5Y clones was assessed using the BrdU incorporation Kit (Roche, Basel, Switzerland). Cells were labeled with BrdU (10 μM BrdU) for 10 h under standard cultivation conditions. The percentage of apoptotic cells in undifferentiated and differentiated (7+7d) SHSY5Y clones was determined using the *in situ* cell death detection Kit (TUNEL, Roche). In both assays, nuclei were counterstained with Hoechst 33342 prior to mounting with Vectashield and imaged with the automated inverted microscope DMI4000B. Ten fields of view per cell clone (20 × magnification) were analyzed by ImageJ software 1.52i (National Institutes of Health, USA). The mean percentage of BrdU^+^ or TUNEL^+^ cells/field of view was calculated (n≥3).

### Statistics

#### Functional *in vitro* analyses

Statistical analyses of functional data are described in S1 Text.

## Supporting information

S1 Text Supplementary methods. (DOCX)

S1 Data Supplementary results. (DOCX)

S1 Table Filtered WES data of patient I. HGVS nomenclature of variants was verified using the batch validation tool Mutalyzer (https://mutalyzer.nl). CADD scores were calculated using the CADD model GRCh37-v1.4 (https://cadd.gs.washington.edu/snv). *For indels, CADD version 1.3 was locally installed. Genes lettered in grey had CADD scores < 13 and were excluded from further analyses. Grey columns were not followed up as no neurological phenotype could be associated to the respective candidate gene after a database (https://geneticassociationdb.nih.gov/; https://www.disgenet.org/) and literature search. Selected candidate genes are highlighted in bold letters. n.a.: not annotated, comp.: compound, AD: Alzheimer disease, ALS: Amyotrophic lateral sclerosis, ID: Intellectual disability, MS: Multiple sclerosis, PD: Parkinson disease.
(PDF)

S2 Table Filtered WES data of patient II. HGVS nomenclature of variants was verified using the batch validation tool Mutalyzer (https://mutalyzer.nl). CADD scores were calculated using the CADD model GRCh37-v1.4 (https://cadd.gs.washington.edu/snv). *For indels, CADD version 1.3 was installed locally. Genes lettered in grey had CADD scores < 13 and were excluded from further analyses. Grey columns were not followed up as no neurological phenotype could be associated to the respective candidate gene after a database (https://geneticassociationdb.nih.gov/; https://www.disgenet.org/) and literature search. Selected candidate genes are highlighted in bold letters. n.a.: not annotated, comp.: compound, AD: Alzheimer disease, ASD: Autism spectrum disorder, BP: Bipolar disorder, ID: Intellectual disability, PD: Parkinson disease, X-ALD: X-linked adrenoleukodystrophy.
(PDF)

S3 Table IPA input (ENS-relevant and HSCR risk genes). (PDF)

S4 Table Rare candidate-specific variants identified in further patients with HSCR. HGVS nomenclature of variants was verified using the batch validation tool Mutalyzer (https://mutalyzer.nl). CADD scores were calculated using the CADD model GRCh37-v1.4 (https://cadd.gs.washington.edu/snv). For gnomAD comparisons, population-matched control cohorts were used (non-Finnish European, 284 patients [8]; East Asian, 443 patients [49]). n.a.: not annotated, EA: South-Asian, EUR: European. Gene isoforms: *ATP7A* NM_001282224; *SREBF1* NM_004176; *ABCD1* NM_000033; *PIAS2* NM_004671. *patient with feeding issues and severe developmental delay.
(PDF)

S5 Table Rare candidate-specific variants identified from clinical exome or genome sequencing data sets. HGVS nomenclature of variants was verified using the batch validation tool Mutalyzer (https://mutalyzer.nl). CADD scores were calculated using the CADD model GRCh37-v1.4 (https://cadd.gs.washington.edu/snv). For gnomAD comparisons control data were used. n.a.: not annotated. Gene isoforms: *ATP7A* NM_000052; *SREBF1* NM_004176; ^#^NM_001005291, *ABCD1* NM_000033; *PIAS2* NM_004671; ^§^NM_173206.
(PDF)

S6 Table Results of protein expression analyses in murine embryonic tissues. Green indicates partial co-expression of both antigens or immunofluorescence signals in close spatial proximity to each other. Red indicates no overlap in respective antigen signals.
(PDF)

S7 Table Summary of validation steps for the four selected candidate genes. (PDF)

S8 Table Oligonucleotides. (PDF)

S9 Table Primary antibodies. (PDF)

S10 Table Secondary antibodies. (PDF)

S11 Table sgRNA. (PDF)

S12 Table Oligonucleotides used for off-target analysis. (PDF)

S13 Table Raw data for figures. (XLSX)

S1 Fig Components of our comparative study pipeline. Flowchart showing the WES analysis approach and the subsequent steps involved in candidate gene A) identification and selection, B) prioritization as well as C) validation. Technical details and cut offs are given. MAF: Minor allele frequency, comp.: compound.
(PDF)

S2 Fig Variant validation in selected candidate genes by Sanger sequencing. Four colored chromatograms are shown for all members of family I (A) and family II (B). In case of heterozygous states, ambiguity codes are given.
(PDF)

S3 Fig Protein expression analyses of candidates in murine embryonic and human fetal tissue sections. (A) Expression analyses of each candidate with the NCC marker Sox10 (green) in murine embryos of stage E9.5 are shown. Sox10+ cells in close proximity to the neck are displayed. Cranial and caudal positions are indicated (Atp7a (A'), Srebf1 (A"), Abcd1 (A"'), Pias2 (A""), all in red). Immunofluorescence signals in close spatial proximity suggesting co-expression, are indicated by white arrowheads. (B) Triple staining in the developing midgut at E13.5 reveals expression of all candidates within or in close spatial proximity to immature neurons (Tubb3 (green)) and immature smooth muscle cells (Sma (cyan)); (Atp7a (B'), Srebf1 (B"), Abcd1 (B"'), Pias2 (B""), all in red) (white arrowheads). The gut lumen is highlighted. (A, B) Bottom images show magnifications of the areas highlighted in the top images. Nuclei were counterstained with Hoechst 33342 (blue). Scale bars: 50 μm. (C) Immunohistochemistry analyses in human fetal colon sections show candidate protein expression in enteric ganglia (marked by a red arrowhead) except for ABCD1 (C"'). Bottom images illustrate the gut epithelial layer where candidates are expressed in enterocytes (ATP7A (C'), SREBF1 (C"), PIAS2 (C""), all in brown). Nuclei were counterstained with hematoxylin (blue). Scale bars: 20 μm.
(PDF)

S4 Fig RNA expression of selected candidates in human fetal colon tissue. Published RNA sequencing data [2] were evaluated for the expression of selected candidates. Bar chart shows reads per kilobase of exon model per million mapped reads (RPKM) in human fetal hindgut specimens of embryonic week (EW) 12, 14, and 16.
(PDF)

S5 Fig CRISPR/Cas9 gene mediated *KO* of *RET* and candidate genes. (A'–D') Gene-specific sgRNAs were designed against marked exons. Cas9-mediated double-strand breaks were repaired by NHEJ causing homozygous or compound heterozygous genome modifications at the respective positions, as verified by Sanger sequencing. Four color chromatograms are shown for the genes (*RET* (A'), *ATP7A* (B'), *SREBF1* (C'), *ABCD1* (D')). Genome editing for *PIAS2* did not work. PAM sites are underscored. Putative Cas9-cutting sites are marked by red arrowheads. (A"–D") *Knockout (KO)* on protein level was validated by Western blot analyses using different protein lysates as internal controls (HEK293TN cells transiently transfected with a gene-specific, tagged overexpression construct, SHSY5Y cells, *mock control* cells) (RET (A"), ATP7A (B"), SREBF1 (C"), ABCD1 (D")). GAPDH was used as a loading control. Predicted protein sizes are annotated. Images show modified blots as individual lanes of respective blots were rearranged if necessary. FL: full length, MAT: mature.
(PDF)

S6 Fig qRT PCR expression profiling of selected cell markers in differentiating cell clones. Gene expression profiles of different cell fate markers such as *P75NTR* and *NES* (neuronal progenitor markers) as well as *UCHL1* and *ASCL1* (advanced neuronal cell fate markers) in differentiating genome-edited cell clones by qRT PCR analysis. (n = 3, mean + standard error of mean; exploratory data analysis by two-sided unpaired t-Test with/without Welch’s correction, *p<0.05; **p<0.01; ***p<0.001).
(PDF)
