# Supplementary material for: A complementary study approach unravels novel players in the pathoetiology of Hirschsprung disease
Source: PLoS Genet. 2020 Nov 5;16(11):e1009106. doi: 10.1371/journal.pgen.1009106 (PMC7643938; doi:10.1371/journal.pgen.1009106)
Supplement: S1 Text — (DOCX) [file pgen.1009106.s001.docx]

**A complementary study approach unravels novel players in the pathoetiology of Hirschsprung disease: S1 Text**

**Supplementary Methods**

**Candidate gene identification** **and selection**

**Whole exome sequencing and variant calling.** The raw reads from each sample were aligned to the 1,000 genome reference sequence (GRCh37 version hs37d5) using BWA aln (version 0.6.2) [1] and duplicate reads were removed with Picard MarkDuplicates (version 1.61) (http://broadinstitute.github.io/picard). The small variants were called together for all the samples in the family using Platypus (version 0.8.1) [2], and gene-based annotations were performed using ANNOVAR for the Gencode gene model (version 19) [3]. Non-coding (5’UTR, 3’UTR and intronic) variants, synonymous mutations, and variants with a minor allele frequency (MAF) >1% in the 1,000 genome phase III [4] or in the Exome Aggregation Consortium database [5] were considered common alleles and discarded, as well as variants detected in 328 whole exome sequencing (WES) or in 177 whole genome sequencing (WGS) local control samples with a frequency above 5%. All SNVs and indels affecting protein sequences as well as variants within ±2 bases around the intron-exon junction were considered functional. Variants were further annotated with several variant-effect prediction tools using dbNSFP (version 2.9) [6]. Next, variants were prioritized according to the disease model (homozygous, hemizygous, compound heterozygous and de novo).

**Transcriptomics analysis in murine embryonic tissue**

**Animals.** All animal experiments were performed in strict compliance with the National Institute of Health’s Guidelines for the Care and Use of Laboratory Animals and were approved according to the EU Directive 2010/63/EU by the Animal Care and Use Committee Germany. Specific pathogen-free animal housing was provided at the Interfacultary Biomedical Faculty, University of Heidelberg, Germany. Mice were fed ad libitum. CD1 wildtype mice were used for all analyses, and the day of vaginal plug was defined as E0.5. For downstream analyses, mice were sacrificed by CO2 asphyxiation.

**Microarray analysis.** Biotinylated antisense cDNA was prepared according to the standard labeling protocol with the GeneChip WT Plus Reagent Kit and the GeneChip hybridization, wash, and stain Kit (both from Thermo Fisher Scientific, Waltham, USA). DNA was hybridized to the chip in a GeneChip Hybridization oven 640, dyed with the GeneChip Fluidics Station 450, and scanned with a GeneChip Scanner 3000. The equipment used was from Affymetrix (Affymetrix, Santa Clara, USA). A Custom CDF Version 22 with ENTREZ-based gene definitions was used to annotate the arrays [7]. The raw fluorescence intensity values were normalized applying quantile normalization and robust multi-array background correction. Data were analyzed at the Microarray-Analytic, Center of Medical Research, Medical Faculty Mannheim of Heidelberg University, Germany. Microarray data (GSE144442) were deposited in the public NCBI GEO database (https://www.ncbi.nlm.nih.gov/geo/).

**Candidate gene validation**

**Sequence validation by Sanger sequencing.** To verify variants identified by WES in selected candidate genes, Sanger sequencing was performed. Oligonucleotides for all sequencing reactions were designed with Primer3 (http://primer3.ut.ee/) and are specified in S8 Table. The HotStar Taq Polymerase Kit (Qiagen) was used for amplicon generation. After PCR product purification using the QIAquick PCR purification Kit (Qiagen), Sanger sequencing was performed at GATC Biotech (Konstanz, Germany).

**IPA network analysis.** Using the Ingenuity Pathway Analysis (IPA) software (Qiagen, Venlo, The Netherlands), 15 networks were generated using a predefined gene list (S3 Table). Next, four networks containing the candidate gene products of interest (ATP7A, SREBF1, ABCD1, PIAS2) were merged. Validated HSCR risk loci based on Luzon-Toro et al. [8], were manually added and connected to the merged network. Subsequently, indirect connections not linked to any candidate gene product were removed and networks further modified as molecules were arranged according to their subcellular localization; non-connected factors were erased.

**Protein expression analyses in murine tissues.** For Immunofluorescence analyses, whole embryos (E9.5, E10.5, E11.5, E13.5) were collected in 1 × PBS (Thermo Fisher Scientific) and fixed in 4% paraformaldehyde (PFA) (Sigma-Aldrich, St. Louis, USA) (in PBS) at 4°C for 24 hours (h) under gentle agitation. Next, embryos were washed twice with 1 × PBS, incubated in 30% sucrose (AppliChem, Darmstadt, Germany) (in DEPC-PBS) (Sigma-Aldrich) at 4°C for 24 h under gentle agitation, then snap frozen in Tissue Freezing Medium (Jung, Leica Biosystems Nussloch GmbH, Germany) in liquid nitrogen, and stored at -80°C until sectioning. Cryosections of 10 µm thickness were prepared using the cryostat microtome CM3050S (Leica Biosystems). For immunofluorescence (IF) staining, whole mount embryonic cryosections were first permeabilized for 7 minutes (min) at room temperature (RT) using either Tween-20 (Carl Roth, Karlsruhe, Germany) or Triton-X100 as detergent (Sigma-Aldrich). Depending on the applied primary antibody, heat-induced antigen retrieval was performed using citrate buffer (1.8 mM citric acid (Merck Millipore, Burlington, USA) + 8.2 mM sodium citrate (AppliChem) + 0.05% Tween-20, pH 6.0) prior to permeabilization. For blocking, sections were incubated with 1% bovine serum albumin (BSA) – fraction V, pH 7.0 (Serva Electrophoresis, Heidelberg, Germany)/10% normal goat serum (NGS) (Thermo Fisher Scientific) (in PBS) supplemented with respective permeabilization detergent for 1 h at RT. Primary antibodies were diluted in blocking solution and incubated overnight (o/n) at 4°C. After washing, secondary antibodies were diluted in corresponding blocking solution and incubated for 1 h at RT. Next, cryosections were washed, counterstained for 4 min with Hoechst 33342 (1:5,000) (Thermo Fisher Scientific) in 1 × PBS and mounted in Vectashield (Vector Laboratories, Peterborough, UK). Triple staining was performed stepwise. Stained sections were imaged using the automated inverted microscope DMI4000B (Leica Biosystems). Primary and secondary antibodies are listed in S9 Table and S10 Table.

**Protein expression analyses in human tissues**

Human fetal FFPE colon tissue sections of 3 µm thickness were generated using the Microm HM 355 rotary microtome (Microm International, Dreieich, Germany). For immunohistochemical (IHC) staining, sections were first deparaffinized and rehydrated. 10 mM EDTA (Sigma-Aldrich) (pH 9.0) was used for heat-induced antigen retrieval. Next, specimens were blocked in antibody diluent solution (Thermo Fisher Scientific) containing 15% NGS (Vector Laboratories). Primary antibody incubation (in blocking solution) was carried out o/n at 4°C. The next day, sections were washed with TBS-T (20 mM TRIS (Sigma-Aldrich) + 137 mM sodium chloride (Sigma-Aldrich), pH 7.6 + 0.5% Tween-20). Secondary antibody incubation using a peroxidase-conjugated antibody diluted in blocking solution was carried out for 1 h at RT. After washing, DAB reaction was performed using the ImmPACT DAB peroxidase substrate Kit (Vector Laboratories) and ddH2O. Nuclear counterstaining was performed with hematoxylin (Carl Roth). After washing, sections were differentiated in running tap water and mounted with Aquatex (Merck Millipore). Staining was imaged using the light microscope BX53 (Olympus, Shinjuku, Japan). Antibodies are described in S9 Table and S10 Table.

**Candidate gene characterization**

**Cloning of candidate gene expression constructs.** Recombinantly expressed candidate gene products in HEK293TN were used as positive controls in immunoblot analyses and generated as follows. The Q5 high fidelity DNA polymerase Kit (New England Biolabs GmbH, Ipswich, USA) was used for insert generation of *RET* and *SREBF1-FL* (full length) overexpression constructs. Oligonucleotides are specified in S8 Table. PCR products were purified using the QIAquick PCR purification Kit. After restriction digest, purified inserts were ligated into the pcDNA3.1-2xFLAG vector backbone using the T4 DNA ligase Kit (Thermo Fisher Scientific).

Plasmids were purified using the GeneJET Plasmid MiniPrep Kit (Thermo Fisher Scientific) or the ZymoPURE II plasmid prep Kit (Zymo Research, Irvine, USA) according to the respective manuals. Plasmids were sequence validated by Sanger sequencing.

pcDNA3.1-2xFLAG-SREBP-1c (#26802) was purchased from Addgene (Watertown, USA), pEGFP-N1-ABCD1 was kindly provided by Dr. Imanaka, University of Toyama (Japan), and pcDNA4-ATP7A-MYC was kindly provided by Bart van de Sluis, University of Groningen (The Netherlands).

**Cell cultivation.** All cells were cultivated at 37°C in a humidified atmosphere (95%) containing 5% CO_2_. SHSY5Y cells (DSMZ, Braunschweig, Germany) were cultured in culture medium (DMEM, 4.5 g/L glucose, sodium pyruvate) supplemented with 1% penicillin/streptomycin (P/S) and 15% fetal bovine serum (FBS)) (all from Thermo Fisher Scientific). HEK293TN cells (Biocompare, San Francisco, USA) were cultured in DMEM (4.5 g/L glucose, sodium pyruvate) supplemented with 10% FBS.

For transfection of HEK293TN cells with gene-specific overexpression constructs, Lipofectamine 2000 (Thermo Fisher Scientific) was used following the manufacturer’s instructions. Transfected cells were harvested for protein isolation after 48 h. Protein lysates served as positive controls in the *knockout* (*KO*) validation analysis.

Neuronal differentiation of SHSY5Y clones was carried out using a modified protocol [9]. For this purpose, 2 × 10^4^ cells/cm^2^ were plated on 0.05 mg/mL collagen I rat tail protein (Col I) (Thermo Fisher Scientific) coated dishes. The next day, medium was changed to culture medium supplemented with 10 µM all-trans retinoic acid (RA) (Sigma-Aldrich). Media was changed again after 3–4 days (d) of cultivation. 7d after RA treatment, cells were first washed twice with DMEM and then differentiation media was added (DMEM (4.5 g/L glucose, sodium pyruvate), 1% P/S, and 50 ng/mL brain-derived neurotrophic factor (BDNF) (Peprotech, Rocky Hill, USA)). Half media changes were performed two times twice a week. Cells were differentiated for an additional 28d (7+28d).

**Design and cloning of sgRNAs for CRISPR/Cas9 genome editing.** To generate candidate-specific SHSY5Y *KO* cell clones, single guide (sg) RNAs were designed using the CCTOP CRISPR/Cas9 target online predictor [10] (S11 Table). Annealed and phosphorylated sgRNA oligonucleotides were cloned into the BbsI (Thermo Fisher Scientific) digested and purified vector backbone pSpCas9(BB)-2A-GFP (Addgene, Watertown, USA) using T4 DNA ligase (Thermo Fisher Scientific). Insert integration was validated by Sanger sequencing.

**CRISPR/Cas9 genome editing of SHSY5Y cells.** SHSY5Y cells were transfected with respective constructs using Lipofectamine 2000 according to the manufacturer’s instructions. For the generation of a *mock control* clone, the empty cloning plasmid was transfected. 48 h after transfection, cells were harvested by trypsinization, cell pellets were resuspended in ice-cold MACS buffer (1 × PBS + 1% FBS + 2.5 mM EDTA), and fluorescent-activated cell sorting (FACS) was performed at the FACS Core Facility of the Department of Medicine V in Heidelberg using a BD FACSAria special order research product cell sorter (BD, Franklin Lakes, USA). Single cells (GFP^+^) were sorted into 96-well plates. Cell clones were expanded in culture medium, replicated, and genotyped. Only clones with a neuronal-like morphology (as defined previously [11]) were replicated.

Genomic DNA was extracted from replicated SHSY5Y clones using the DirectPCR lysis reagent for cells (VWR, Radnor, USA) following the manufacturer’s instructions. Oligonucleotides used for genotyping are listed in S8 Table. The pSTBlue-1 AccepTor Vector Kit (Merck Millipore) was used to separate the different alleles of genome-edited clones presenting with a compound heterozygous state according to the manufacturer’s instructions. PCR products for Sanger sequencing were generated with the HotStar polymerase Kit and purified using the QIAquick PCR purification Kit (both from Qiagen). For off-target analysis, the predicted top 20 off-target sites generated by the CCTOP CRISPR/Cas9 target online predictor were further filtered for exonic off-target sites. In addition, putatively essential intra-/intergenic off-target sites encoding e.g., non-coding RNAs or containing enhancer sites were identified using the UCSC Genome browser. Oligonucleotides specific to off-target sites are shown in S12 Table.

Gene-specific *KO* clones were validated for absent protein expression by Western blot analyses, as described below.

***KO* validation on protein level.** For *KO* validation on the protein level, cell pellets of edited cell clones or transfected HEK293TN cells were lysed in Radio Immuno Precipitation Assay buffer (150mM sodium chloride (Sigma-Aldrich)) + 1% Igepal (Santa Cruz Biotechnologies, Dallas, USA) + 0.5% sodium deoxycholate + 0.1% sodium dodecyl sulfate (SDS) + 50 mM Tris (pH 8.0, all from Sigma-Aldrich) supplemented with a proteinase and phosphatase inhibitor cocktail (Thermo Fisher Scientific). The protein concentration was assessed using the Pierce BCA protein assay Kit (Thermo Fisher Scientific) according to the manual. 5–50 µg of total protein was separated on an 8% or 10% SDS polyacrylamide gel or on a NuPAGE 4–12% Bis-Tris gradient gel (Thermo Fisher Scientific). Proteins were transferred onto a PVDF Immobilon-FL membrane (Merck Millipore). For imaging, the Odyssey system (LI-COR, Lincoln, USA) was used. Antibodies are described in S9 Table and S10 Table.

**Expression analyses in genome-edited clones**

**qPCR.** For mRNA expression analyses in SHSY5Y clones, cells were harvested and collected in TRIzol. For RNA isolation, TRIzol specimens were thawed on ice and 200 µL of chloroform (Sigma-Aldrich)/mL were added. Next, samples were mixed vigorously, incubated for 5 min at RT, and centrifuged for 15 min at 4°C at full speed. The aqueous phase was transferred into a new tube and mixed with 0.5 volumes of ethanol (Sigma-Aldrich). Corresponding to the instructions of the RNAqueous-Micro Total RNA Isolation Kit (Thermo Fisher Scientific), 150 µl of the lysate/ethanol mixture were transferred onto a micro filter cartridge. All further steps were carried out according to the supplier’s instructions.

800 ng of total RNA was used for cDNA synthesis using the SuperScript III first strand synthesis system (Thermo Fisher Scientific) (ratio random hexamer/Oligo(dT) primers - 1:1). Quantitative real time expression analysis (qRT PCR) was performed using the SYBR Green Lo-ROX Fast Mix (Bioline Meridian Bioscience, Cincinnati, USA) according to the manufacturer’s instructions. qRT PCR analysis was performed in duplicate (n=3). Relative mRNA expression levels were calculated by the relative standard curve method via normalization to the expression of the two reference genes SDHA and GAPDH. Oligonucleotide sequences are listed in S8 Table.

**Immunofluorescence.** For IF staining of differentiated clones, cells were fixed in 4% PFA (in PBS) for 20 min at RT. Next, specimens were blocked for 1 h at RT in 1% BSA/10% NGS (in PBS). Primary antibodies were diluted in blocking solution and cells were incubated for 1 h at RT. After washing, secondary antibodies diluted in blocking solution were applied and incubated for 1 h at RT. Counterstaining was performed using the nuclear dye Hoechst 33342 for 4 min (1:5,000). Samples were mounted with AquaPolymount (Polysciences, Hirschberg, Germany). Imaging was performed using the automated inverted microscope DMI4000B. Antibodies are given in S9 Table and S10 Table.

**Cell migration assay.** Cell migration of undifferentiated and differentiated (7+1d) SHSY5Y cell clones was investigated by Boyden chamber assays. 6.5-mm transwell inserts (5 µm pore size/polycarbonate membrane) (Corning, Corning, USA) were coated with 0.05 mg/mL Col I. Thereafter, 1.2 × 10^5^ cells (undifferentiated) or 1.5 × 10^5^ cells (differentiated) were seeded on inserts in serum-free media (DMEM high glucose). To measure the directed migratory behavior, lower compartments were either filled with culture media/differentiation media (FBS/BDNF as chemoattractant; positive control) or DMEM high glucose only (no chemoattractant; negative control). 24 h after incubation under standard conditions, cells from the inside of the inserts were removed using 1 × PBS-wetted cotton swabs. Migrated cells at the opposite site were fixed in ice-cold methanol (Sigma-Aldrich) for 20 min at RT, washed with 1 × PBS, and counterstained with Hoechst 33342. Membranes were mounted in Vectashield and imaged at five spots (20 × magnification) equally distributed over the membrane using the automated inverted microscope DMI4000B. Nuclei were counted using the ImageJ software 1.52i (National Institutes of Health, USA) and the mean cell number/membrane was calculated (n≥3).

**Statistics**

**Evaluation of functional *in vitro* analyses.** An exploratory data analysis was carried out for qRT PCR expression data. For this purpose, mean expression levels for a specific gene and an individual time point were compared between the *mock control* clone and each of the gene-specific SHSY5Y *KO* clones using a two-sided unpaired t-Test (with Welch’s correction in case of significantly different standard deviations of the compared two means). For calculation, the GraphPad Prism Software 8.0 (GraphPad, San Diego, USA) was used. Corresponding to this analysis, statistical results are purely descriptive.

Data of functional *in vitro* assays (Boyden Chamber migration, cell proliferation or cell death detection assay) was tested for statistical significance using the GraphPad Prism Software 8.0. Mean values were compared between the *mock control* clone and each of the gene-specific *KO* clones using a one-way ANOVA. Results were Bonferroni-corrected for multiple testing against the number of pairwise comparisons.

**References**

1. Li H, Durbin R. Fast and accurate long-read alignment with Burrows-Wheeler transform. Bioinformatics. 2010;26(5):589-95.

2. Rimmer A, Phan H, Mathieson I, Iqbal Z, Twigg SRF, Wilkie AOM, et al. Integrating mapping-, assembly- and haplotype-based approaches for calling variants in clinical sequencing applications. Nature genetics. 2014;46(8):912-8.

3. Frankish A, Diekhans M, Ferreira AM, Johnson R, Jungreis I, Loveland J, et al. GENCODE reference annotation for the human and mouse genomes. Nucleic acids research. 2019;47(D1):D766-d73.

4. Auton A, Brooks LD, Durbin RM, Garrison EP, Kang HM, Korbel JO, et al. A global reference for human genetic variation. Nature. 2015;526(7571):68-74.

5. Lek M, Karczewski KJ, Minikel EV, Samocha KE, Banks E, Fennell T, et al. Analysis of protein-coding genetic variation in 60,706 humans. Nature. 2016;536(7616):285-91.

6. Liu X, Jian X, Boerwinkle E. dbNSFP v2.0: a database of human non-synonymous SNVs and their functional predictions and annotations. Hum Mutat. 2013;34(9):E2393-402.

7. Dai M, Wang P, Boyd AD, Kostov G, Athey B, Jones EG, et al. Evolving gene/transcript definitions significantly alter the interpretation of GeneChip data. Nucleic acids research. 2005;33(20):e175.

8. Luzón-Toro B, Gui H, Ruiz-Ferrer M, Sze-Man Tang C, Fernandez RM, Sham PC, et al. Exome sequencing reveals a high genetic heterogeneity on familial Hirschsprung disease. Scientific reports. 2015;5:16473.

9. Encinas M, Iglesias M, Liu Y, Wang H, Muhaisen A, Cena V, et al. Sequential treatment of SH-SY5Y cells with retinoic acid and brain-derived neurotrophic factor gives rise to fully differentiated, neurotrophic factor-dependent, human neuron-like cells. Journal of neurochemistry. 2000;75(3):991-1003.

10. Stemmer M, Thumberger T, Del Sol Keyer M, Wittbrodt J, Mateo JL. CCTop: An Intuitive, Flexible and Reliable CRISPR/Cas9 Target Prediction Tool. PloS one. 2015;10(4):e0124633.

11. Ross RA, Spengler BA, Biedler JL. Coordinate morphological and biochemical interconversion of human neuroblastoma cells. J Natl Cancer Inst. 1983;71(4):741-7.
