# Supplementary material for: A complementary study approach unravels novel players in the pathoetiology of Hirschsprung disease
Source: PLoS Genet. 2020 Nov 5;16(11):e1009106. doi: 10.1371/journal.pgen.1009106 (PMC7643938; doi:10.1371/journal.pgen.1009106)
Supplement: S1 Data — (DOCX) [file pgen.1009106.s002.docx]

**A complementary study approach unravels novel players in the pathoetiology of Hirschsprung disease: S1 Data**

**Supplementary Results**

**Candidate gene validation and characterization by expression analyses**

All candidates were expressed in or in close spatial proximity to pre-migratory vagal neural crest cells (NCCs) at stage E9.5, as demonstrated by co-staining with Sox10 (S3A Fig). In the developing midgut at E13.5, expression in immature neurons and smooth muscle cells or in their respective microenvironment was detected by co-labeling with Tubb3 (neuronal marker) and Sma (muscle marker) (S3B Fig), respectively (S7 Table, S1 Fig). Complementary IHC analyses of human fetal colon specimens (25^th^ week of gestation) (S3C Fig) confirmed a prominent expression in enteric ganglia for ATP7A (S3C’ Fig) and PIAS2 (S3C’’’’ Fig). In contrast, SREBF1 (S3 C’’ Fig) was only marginally expressed and ABCD1 staining was not detected (S3C’’’ Fig). However, all candidates were expressed in the gut mucosa at this stage. Specifically, ATP7A (S3C’ Fig) was found in most cells of the gut epithelium and the lamina propria while SREBF1 (S3C’’ Fig) was distinctly expressed in individual cells at the crypt’s base. ABCD1 (S3 C’’’ Fig) showed an almost ubiquitous expression, presumably in immune cells within the lamina propria, while PIAS2-specific signals were observed in cells located at the base of the crypts (S3C’’’’ Fig) (S7 Table, S1 Fig).

**Determining the neuronal specific role of candidates**

**Generation of gene-specific *KO* clones.** Off-target effects of all *KO* clones was excluded. The *SREBF1 KO* clone harbored an unintended heterozygous one-nucleotide insertion in intron 7 of *EEF1A2*. Detailed *in silico* analyses using ENCODE data [1] demonstrated that the introduced nucleotide did not reside in the annotated EST; furthermore, the annotated DNase cluster at this side has not been reported yet in the SHSY5Y cell line (off-target side in *EEF1A2* (Chr20:63488553-63488575, GRCh38/hg38); ENCODE data accessible under: <https://genome.ucsc.edu/>). Therefore, relevance of this off-target effect was considered unlikely.

**Expression analysis of SHSY5Y cell clones.** Expression of the neuronal progenitor marker *P75NTR* increased in the *mock control*, the *RET KO*, and the *SREBF1 KO* clones during early stages of neuronal differentiation while at later stages, gene activity was downregulated. The *ATP7A KO* clone displayed the lowest and almost stable *P75NTR* expression throughout differentiation. In contrast, the *ABCD1 KO* clone showed the highest *P75NTR* expression, with peak levels at 7+14d. Significant differences were only detectable for the *ABCD1 KO* and the *ATP7A KO* clones during later differentiation, 7+28d (S6 Fig).

*NES* expression was higher in the *ABCD1 KO* clone than all other clones. Specifically, *NES* expression decreased during early differentiation stages before peaking at 7+14d. At 7d, expression was significantly higher than in the *mock control* clone. Slightly increasing expression values from the undifferentiated to the 7+28d differentiated state were observed in all other clones although fluctuations of different degrees were detected during differentiation. Significantly decreased expression levels were detected for the *ATP7A KO* clone after 7+14d (S6 Fig).

Expression of the pan-neuronal marker *UCHL1* increased constantly during differentiation in the *mock control* clone. Expression was slightly lower in the *SREBF1 KO* clone, but showed comparable regulation. In contrast, for the *RET KO* and the *ATP7A KO* clones, expression levels first decreased before they increased after 7+7d of differentiation. For the *ABCD1 KO* clone, a comparable regulation pattern was observed although relative expression levels were higher throughout differentiation. All edited clones showed significant differences at different stages of neuronal maturation (S6 Fig).

A drastic decrease in *ASCL1* expression from the undifferentiated to the 7d differentiated state was observed in in all clones. The *ATP7A KO* clone displayed only minimal expression of this marker at later differentiation states while expression increased until 7+7d in all other clones, before being downregulated with progressing differentiation. Significant differences in *ASCL1* expression were detected in *RET KO* and the *ATP7A KO* clones after 7d (S6 Fig).

**References**

1. Rosenbloom KR, Sloan CA, Malladi VS, Dreszer TR, Learned K, Kirkup VM, et al. ENCODE data in the UCSC Genome Browser: year 5 update. Nucleic acids research. 2013;41(Database issue):D56-63.
