# Supplementary material for: A complementary study approach unravels novel players in the pathoetiology of Hirschsprung disease
Source: PLoS Genet. 2020 Nov 5;16(11):e1009106. doi: 10.1371/journal.pgen.1009106 (PMC7643938; doi:10.1371/journal.pgen.1009106)
Supplement: S1 Table — HGVS nomenclature of variants was verified using the batch validation tool Mutalyzer (https://mutalyzer.nl). CADD scores were calculated using the CADD model GRCh37-v1.4 (https://cadd.gs.washington.edu/snv). *For indels, CADD version 1.3 was locally installed. Genes lettered in grey had CADD scores < 13 and were excluded from further analyses. Grey columns were not followed up as no neurological phenotype could be associated to the respective candidate gene after a database (https://geneticassociationdb.nih.gov/; https://www.disgenet.org/) and literature search. Selected candidate genes are highlighted in bold letters. n.a.: not annotated, comp.: compound, AD: Alzheimer disease, ALS: Amyotrophic lateral sclerosis, ID: Intellectual disability, MS: Multiple sclerosis, PD: Parkinson disease. (PDF) [file pgen.1009106.s003.pdf]

S1 Table: Filtered WES data of patient I

HGVS nomenclature of variants was verified using the batch validation tool Mutalyzer (https://mutalyzer.nl). CADD scores were calculated using the CADD model GRCh37-v1.4 (https://cadd.gs.washington.edu/snv). *For indels, CADD version 1.3 was locally installed. Genes lettered in grey were excluded from further analyses as they had either CADD scores < 13 or no neurological phenotype could be associated to the respective candidate gene after a database (https://geneticassociationdb.nih.gov/; https://www.disgenet.org/) and literature search. Selected and followed up candidate genes are highlighted in bold letters. n.a.: not annotated, comp.: compound, AD: Alzheimer disease, ALS: Amyotrophic lateral sclerosis, ID: Intellectual disability, MS: Multiple sclerosis, PD: Parkinson disease.

Gene name	Nucleotide/Amino acid change	Mutation type	CADD score	Associated neurological phenotypes
ATP7A	**ENST00000341514.6: c.4067G>A; p.R1356Q**	**hemizygous**	**26.9**	**Menkes disease, ID, Epilepsy, AD, mental retardation, ALS, dementia, PD, Peripheral neuropathy**
DNAH14	ENST00000439375.2: c.4857_4860del; p.1619_1620del	comp. heterozygous	36*	n.a.
DNAH14	ENST00000445597.2: c.356G>A;p.G119D,		8.636	
DSG1	ENST00000462981.2 c.192C>T; p.Y64Y	comp. heterozygous	23.6	AD
DSG1	ENST00000462981.2: c.685G>A; p.G229S		24	
KIAA1522	ENST00000401073.2: c.68G>T; p.R23L	*de novo*	23.2	n.a.
PHF16	ENST00000424392.1: c.409C>T; p.R137C	hemizygous	26.9	n.a.
PTPRK	ENST00000368227.3: c.2564G>A; p.R855H	comp. heterozygous	22.6	n.a.
PTPRK	ENST00000368227.3: c.1929G>T; p.K643N		25.8	
PVRL2	ENST00000252485.4: c.227C>T; p.P76L	*de novo*	12.23	AD, MS
SREBF1	**ENST00000261646.5: c.3172G>C; p.D1058H**	**comp. heterozygous**	**25.5**	**AD, Dementia, PD, Schizophrenia**
SREBF1	**ENST00000261646.5: c.2435G>A; p.R812Q**		**18.62**	
STAG2	ENST00000455404.1: c.116A>G; p.K39R	hemizygous	23.5	ID
STRC	ENST00000541030.1: c.2599C>T; p.L867F	comp. heterozygous	24.7	n.a.
STRC	ENST00000541030.1: c.2242C>T; p.R748W		23.7	
ZNF275	ENST00000421401.3: c.877T>C; p.Y293H	hemizygous	23.5	n.a.
