# Supplementary material for: A complementary study approach unravels novel players in the pathoetiology of Hirschsprung disease
Source: PLoS Genet. 2020 Nov 5;16(11):e1009106. doi: 10.1371/journal.pgen.1009106 (PMC7643938; doi:10.1371/journal.pgen.1009106)
Supplement: S2 Table — HGVS nomenclature of variants was verified using the batch validation tool Mutalyzer (https://mutalyzer.nl). CADD scores were calculated using the CADD model GRCh37-v1.4 (https://cadd.gs.washington.edu/snv). *For indels, CADD version 1.3 was installed locally. Genes lettered in grey had CADD scores < 13 and were excluded from further analyses. Grey columns were not followed up as no neurological phenotype could be associated to the respective candidate gene after a database (https://geneticassociationdb.nih.gov/; https://www.disgenet.org/) and literature search. Selected candidate genes are highlighted in bold letters. n.a.: not annotated, comp.: compound, AD: Alzheimer disease, ASD: Autism spectrum disorder, BP: Bipolar disorder, ID: Intellectual disability, PD: Parkinson disease, X-ALD: X-linked adrenoleukodystrophy. (PDF) [file pgen.1009106.s004.pdf]

S2 Table: Filtered WES data of patient II

HGVS nomenclature of variants was verified using the batch validation tool Mutalyzer (https://mutalyzer.nl). CADD scores were calculated using the CADD model GRCh37-v1.4 (https://cadd.gs.washington.edu/snv). *For indels, CADD version 1.3 was installed locally. Genes lettered in grey were excluded from further analyses as they had either CADD scores < 13 or no neurological phenotype could be associated to the respective candidate gene after a database (https://geneticassociationdb.nih.gov/; https://www.disgenet.org/) and literature search. Selected and followed up candidate genes are highlighted in bold letters. n.a.: not annotated, comp.: compound, AD: Alzheimer disease, ASD: Autism spectrum disorder, BP: Bipolar disorder, ID: Intellectual disability, PD: Parkinson disease, X-ALD: X-linked adrenoleukodystrophy.

Gene name	Nucleotide/Amino acid change	Mutation type	CADD score	Associated neurological phenotypes
ABCD1	**ENST00000218104.3: c.41C>G; p.T14R**	**hemizygous**	**15.38**	**X-ALD, AD, Dementia, Epilepsy, ID, Peripheral neuropathy, Psychotic disorders**
CNKSR2	ENST00000425654.2: c.2588A>C; p.N863T	hemizygous	18.93	Epilepsy, ID, Mental retardation
EBP	ENST00000495186.1: c.511C>T; p.R171C	hemizygous	21.6	ID, epilepsy. Mental retardation, Dementia, AD, Schizophrenia
FER1L6	ENST00000522917.1: c.2827G>T; p.G943W	comp. heterozygous	33	n.a.
FER1L6	ENST00000522917.1: c.2981T>G; p.V994G		28.4	
MXRA5	ENST00000217939.6: c.1783G>A; p.V595M	hemizygous	15.17	ID, ASD
OR5P2	ENST00000329434.2: c.56G>A; p.G19D	hemizygous	23.7	n.a.
PIAS2	**ENST00000585916.1: c.889C>T; p.R297W**	***de novo***	**26.9**	**PD**
RNF213	ENST00000456466.1: c.1637C>T; p.T546I	comp. heterozygous	8.843	ID, mental retardation, epilepsy
RNF213	ENST00000582970.1: c.6979A>G; p.N2327D		4.350	
SLC2A4	ENST00000571308.1: c.236C>G; p.T79S	homozygous	14.96	AD
SPTA1	ENST00000368147.4: c.6927G>T; p.M2309I	comp. heterozygous	25.2	ASD
SPTA1	ENST00000368147.4: c.4564A>G; p.T1522A		17.61	
SYNE2	ENST00000394768.2: c.4907T>C; p.I1636T	comp. heterozygous	16.08	ASD
SYNE2	ENST00000441438.2: c.488A>T; p.Q163L		14.60	
TENM4	ENST00000278550.7: c.5729C>T; p.A1910V	*de novo*	21.3	ASD, BP, Schizophrenia

TFDP3	ENST00000310125.4: c.427G>A; p.A143T	hemizygous	7.926	n.a.
VSIG4	ENST00000412866.2: c.866C>T; p.T289I	hemizygous	13.84	n.a.
VSIG4	ENST00000455586.2: c.274G>T; p.V92F	hemizygous	14.96	n.a.
