# Supplementary material for: A complementary study approach unravels novel players in the pathoetiology of Hirschsprung disease
Source: PLoS Genet. 2020 Nov 5;16(11):e1009106. doi: 10.1371/journal.pgen.1009106 (PMC7643938; doi:10.1371/journal.pgen.1009106)
Supplement: S3 Table — (PDF) [file pgen.1009106.s005.pdf]

S3 Table: IPA input (ENS-relevant and HSCR risk genes)

Gene	Gene ID	Gene	Gene ID	Gene	Gene ID
ALDH1A2	ENSG00000128918	*HOXA4*	ENSG00000197576	*RBP4*	ENSG00000138207
ARHGEF3	ENSG00000163947	*HOXB5*	ENSG00000120075	*RET*	ENSG00000165731
ARTN	ENSG00000117407	*HOXD4*	ENSG00000170166	*RMRP*	ENSG00000277027
ASCL-1	ENSG00000139352	*IFNGR2*	ENSG00000159128	*SALL4*	ENSG00000101115
BBS1-11	ENSG00000174483	*IHH*	ENSG00000163501	*SCG3*	ENSG00000104112
CADM1	ENSG00000182985	*IKBKAP*	ENSG00000070061	*SEMA3A*	ENSG00000075213
CARTPT	ENSG00000164326	*IL10RB*	ENSG00000243646	*SEMA3C*	ENSG00000075223
CBR1	ENSG00000159228	*ITGB1*	ENSG00000150093	*SEMA3D*	ENSG00000153993
CDC42	ENSG00000070831	*JAG1*	ENSG00000101384	*SERPINI1*	ENSG00000163536
CDH2	ENSG00000170558	*JAG2*	ENSG00000184916	*SHH*	ENSG00000164690
CRMP1	ENSG00000072832	*KIAA1279*	ENSG00000198954	*SLC6A2*	ENSG00000103546
CSTB	ENSG00000160213	*KIF26A*	ENSG00000066735	*SMO*	ENSG00000128602
CTNNAL1	ENSG00000119326	*KLF4*	ENSG00000136826	*SOD1*	ENSG00000142168
DCC	ENSG00000187323	*L1CAM*	ENSG00000198910	*SON*	ENSG00000159140
DCX	ENSG00000077279	*LGI4*	ENSG00000153902	*SOX10*	ENSG00000100146
DHCR7	ENSG00000172893	*MAB21L1*	ENSG00000180660	*SOX2*	ENSG00000181449
DICER1	ENSG00000100697	*MAPK10*	ENSG00000109339	*SOX8*	ENSG00000005513
DLL1	ENSG00000198719	*MAPT*	ENSG00000186868	*SPRY2*	ENSG00000136158
DLL3	ENSG00000090932	*MLLT11*	ENSG00000213190	*STMN2*	ENSG00000104435
DLX1	ENSG00000144355	*NAV2*	ENSG00000166833	*STMN3*	ENSG00000197457
DNMT3B	ENSG00000088305	*NKX2-1*	ENSG00000136352	*SUFU*	ENSG00000107882
DPYSL3	ENSG00000113657	*NOG*	ENSG00000183691	*SYT11*	ENSG00000132718
EBF3	ENSG00000108001	*NOTCH1*	ENSG00000148400	*TAGLN3*	ENSG00000144834
ECE1	ENSG00000117298	*NOTCH2*	ENSG00000134250	*TBX3*	ENSG00000135111
EDN3	ENSG00000124205	*NOTCH3*	ENSG00000074181	*TCF4*	ENSG00000196628
EDNRB	ENSG00000136160	*NRG1*	ENSG00000157168	*TCF7L2*	ENSG00000148737
ELAVL2	ENSG00000107105	*NRG3*	ENSG00000185737	*TCOF1*	ENSG00000070814
ELAVL4	ENSG00000162374	*NRP1*	ENSG00000099250	*TFAM*	ENSG00000108064
ERBB2	ENSG00000141736	*NRTN*	ENSG00000171119	*TFF3*	ENSG00000160180
ERBB3	ENSG00000065361	*NTF3*	ENSG00000185652	*TGFB2*	ENSG00000092969
ERBB4	ENSG00000178568	*NTRK3*	ENSG00000140538	*TLX2*	ENSG00000115297
ERCC1	ENSG00000012061	*PAX3*	ENSG00000135903	*TMEFF2*	ENSG00000144339

ETV1	ENSG00000006468	*PCDHA1*	ENSG00000204970	*TPH2*	ENSG00000139287
FGF13	ENSG00000129682	*PDS5A*	ENSG00000121892	*TREX1*	ENSG00000213689
GAP43	ENSG00000172020	*PDS5B*	ENSG00000083642	*TTC3*	ENSG00000182670
GDNF	ENSG00000168621	*PFKL*	ENSG00000141959	*TUBB3*	ENSG00000258947
GFRA1	ENSG00000151892	*PHACTR4*	ENSG00000204138	*UCHL1*	ENSG00000154277
GFRA2	ENSG00000168546	*PHOX2A*	ENSG00000165462	*VIP*	ENSG00000146469
GFRA3	ENSG00000146013	*PHOX2B*	ENSG00000109132	*ZEB2*	ENSG00000169554
GFRA4	ENSG00000125861	*PLXNA1*	ENSG00000114554	*ZIC2*	ENSG00000043355
GLI1	ENSG00000111087	*PLXNB1*	ENSG00000164050		
GLI2	ENSG00000074047	*POFUT1*	ENSG00000101346		
GLI3	ENSG00000106571	*PROK1*	ENSG00000143125		
GNG2	ENSG00000186469	*PROK2*	ENSG00000163421		
GNG3	ENSG00000162188	*PROKR1*	ENSG00000169618		
GRB10	ENSG00000106070	*PROKR2*	ENSG00000101292		
HAND2	ENSG00000164107	*PRPH*	ENSG00000135406		
HES1	ENSG00000114315	*PSPN*	ENSG00000125650		
HLX	ENSG00000136630	*PTCH1*	ENSG00000185920		
HMP19	ENSG00000170091	*PTEN*	ENSG00000171862		
HMX3	ENSG00000188620	*RAC1*	ENSG00000136238		
