# Supplementary material for: A complementary study approach unravels novel players in the pathoetiology of Hirschsprung disease
Source: PLoS Genet. 2020 Nov 5;16(11):e1009106. doi: 10.1371/journal.pgen.1009106 (PMC7643938; doi:10.1371/journal.pgen.1009106)
Supplement: S4 Table — HGVS nomenclature of variants was verified using the batch validation tool Mutalyzer (https://mutalyzer.nl). CADD scores were calculated using the CADD model GRCh37-v1.4 (https://cadd.gs.washington.edu/snv). For gnomAD comparisons, population-matched control cohorts were used (non-Finnish European, 284 patients [8]; East Asian, 443 patients [49]). n.a.: not annotated, EA: South-Asian, EUR: European. Gene isoforms: ATP7A NM_001282224; SREBF1 NM_004176; ABCD1 NM_000033; PIAS2 NM_004671. *patient with feeding issues and severe developmental delay. (PDF) [file pgen.1009106.s006.pdf]

S4 Table: Rare candidate-specific variants identified in further patients with HSCR

HGVS nomenclature of variants was verified using the batch validation tool Mutalyzer (https://mutalyzer.nl). CADD scores were calculated using the CADD model GRCh37-v1.4 (https://cadd.gs.washington.edu/snv). For gnomAD comparisons, population-matched control cohorts were used (non-Finnish European, 324 patients [1]; East Asian, 443 patients [2]). n.a.: not annotated, EA: South-Asian, EUR: European. Gene isoforms: *ATP7A* NM_001282224; *SREBF1* NM_004176; *ABCD1* NM_000033; *PIAS2* NM_004671. *patient with feeding issues and severe developmental delay

Candidate	Variant		CADD scores	Number of affected carriers	gnomAD comparison allele count/allele number (frequency)	Patient cohort	Variant in Baylor Data Clinical WES/WGS
	Nucleotide change	Amino acid change					
ATP7A	c.3649C>T	p.R1217W	24.2	1	6/30958 (0.0001938)	EUR	no
	c.3697A>G	p.M1233V	24.6	2	2/34845 (0.00005740)		no
	c.1493C>G	p.T498S	25.9	1	1/6915 (0.0001446)	EA	no
	c.1756C>G	p.L586V	20.6	1	2/6917 (0.0002891)		no
	c.2059A>C	p.M687L	14.04	1	n.a.		no
	c.2878G>A	p.V960I	22.2	1	n.a.		no
SREBF1	c.85A>C	p.I29L	23.8	1	n. a.	EUR	no
	c.2213C>G	p.T738R	25.4	1	n. a.		no
	c.2435G>A	p.R812Q	18.62	2	259/48276 (0.005365)		no
	c.3370G>A	p.D1124N	33	1	n. a.		no
	c.88G>C	p.E30Q	29.3	1	n. a.	EA	no
	c.122G>A	p.S41N	15.83	1	n. a.		no
	c.655G>T	p.A219S	15.64	1	n. a.		no
	c.1475C>T	p.T492M	20.8	2	7/9468 (0.0007393)		yes*
	c.1735G>A	p.A579T	23.4	1	n. a.		no
	c.2594C>T	p.T865I	22.7	1	n. a.		no
ABCD1	c.2033G>A	p.G678D	26.5	4	1/20983 (0.00003228)	EUR	no
	c.1126G>C	p.E376Q	22	1	1/6917 (0.0001446)	EA	no
PIAS2	c.236A>G	p.K79R	16.05	2	0/42766	EUR	no
	c.946C>T	p.P316S	23	1	1/12346 (0.000081)	EA	no
	c.1522C>T	p.Q508*	44	1	n. a.		no
	c.1627A>G	p.S543G	22	1	n. a.		no

	c.1777A>G	p.T593A	16.65	1	12/9042 (0.001327)		no

References

1. Tilghman JM, Ling AY, Turner TN, Sosa MX, Krumm N, Chatterjee S, et al. Molecular Genetic Anatomy and Risk Profile of Hirschsprung's Disease. N Engl J Med. 2019;380(15):1421-32.
2. Tang CS, Li P, Lai FP, Fu AX, Lau ST, So MT, et al. Identification of Genes Associated With Hirschsprung Disease, Based on Whole-Genome Sequence Analysis, and Potential Effects on Enteric Nervous System Development. Gastroenterology. 2018;155(6):1908-22.e5.
