# Supplementary material for: A complementary study approach unravels novel players in the pathoetiology of Hirschsprung disease
Source: PLoS Genet. 2020 Nov 5;16(11):e1009106. doi: 10.1371/journal.pgen.1009106 (PMC7643938; doi:10.1371/journal.pgen.1009106)
Supplement: S5 Table — HGVS nomenclature of variants was verified using the batch validation tool Mutalyzer (https://mutalyzer.nl). CADD scores were calculated using the CADD model GRCh37-v1.4 (https://cadd.gs.washington.edu/snv). For gnomAD comparisons control data were used. n.a.: not annotated. Gene isoforms: ATP7A NM_000052; SREBF1 NM_004176; #NM_001005291, ABCD1 NM_000033; PIAS2 NM_004671; §NM_173206. (PDF) [file pgen.1009106.s007.pdf]

S5 Table: Rare candidate-specific variants identified from clinical exome or genome sequencing data sets

HGVS nomenclature of variants was verified using the batch validation tool Mutalyzer (https://mutalyzer.nl). CADD scores were calculated using the CADD model GRCh37-v1.4 (https://cadd.gs.washington.edu/snv). For gnomAD comparisons control data were used. n.a.: not annotated. Gene isoforms: *ATP7A* NM_000052; *SREBF1* NM_004176; #NM_001005291, *ABCD1* NM_000033; *PIAS2* NM_004671; §NM_173206.

**Candidate**	**Variant**				
	**Nucleotide change**	**Amino acid change**	**CADD scores**	**gnomAD comparison allele count/allele number (frequency)**	**Associated GI and/or CNS phenotypes**
ATP7A	c.119A>C	p.K40T	23.7	2/87913 (0,00002275)	seizure, failure to thrive
	c.121G>A	p.V41I	25.7	1/7533 (0,00013275)	global developmental delay, autistic spectrum disorder, seizures, autonomic neuropathy, gastroesophageal reflux
	c.202G>A	p.D68N	24.4	2/77183 (0,00002591)	gross motor and speech delay, optic nerve hypoplasia, failure to thrive
	c.239C>T	p.P80L	23	45/79672 0,00056482	delayed motor milestones, delayed speech, failure to thrive, neuropathic process with denervation atrophy
	c.314G>A	p.S105N	15.78	n.a.	severe delayed motor milestones, absent speech, seizures, failure to thrive
	c.317C>T	p.T106I	15.83	1/7660 0,00013055	delayed motor milestone, delayed speech, autism, failure to thrive
	c.617T>G	p.L206R	27	n.a.	feeding difficulties
	c.729G>T	p.K243N	16.44	1/80325 0,00001245	failure to thrive in infancy, hyperreflexia
	c.880A>T	p.N294Y	24.2	14/88050 0,00015900	failure to thrive
	c.1032_1033del	p.R344fs	32	n.a.	delayed motor milestones, multifocal seizures from both left and right hemispheres
	c.1037G>T	p.S346I	22.7	n.a.	moderate intellectual disability, chronic abdominal pain, gastroesophageal reflux
	c.1406A>G	p.E469G	17.01	n.a.	seizures, failure to thrive
	c.1474A>G	p.I492V	15.47	1/80318 0,00001245	delayed motor milestones, delayed speech, intellectual disability, failure to thrive

	c.1504T>C	p.C502R	27.7	3/88036<br>0,00003408	developmental regression, speech delay, failure to thrive, structural brain abnormalities
	c.1941A>G	p.I647M	24.2	7/80325<br>0,00008715	failure to thrive, esophagitis, microcephaly
	c.1996G>C	p.G666R	28.2	n.a.	global developmental delay, microcephaly, generalized tonic-clonic seizures
	c.2149T>G	p.F717V	24.5	n.a.	tracheoesophageal fistula with esophageal atresia, irritable bowel syndrome
	c.2225A>G	p.K742R	16.51	3/80332<br>0,00003735	appendicular rigidity, hyperekplexia, frequent falls and dropping of cups and spoons, blurred and stuttered speech
	c.2254C>G	p.L752V	23.7	2/88008<br>0,00002273	delayed motor milestones, delayed speech, intellectual disability, intractable epilepsy
	c.2313G>C	p.E771D	22.2	1/80347<br>0,00001245	delayed motor milestones, failure to thrive, suspected visual and hearing deficit, cerebellar hypoplasia
	c.2519A>T	p.E840V	27.4	14/88000<br>0,00015909	seizures, global developmental delay, feeding difficulties
	c.2530C>T	p.R844C	30	12/80321<br>0,00014940	delayed motor milestones, delayed speech, intellectual disability, seizure disorder, microcephaly, failure to thrive, severe brain atrophy, small cerebellum
	c.2530C>T	p.R844C	30	12/80321<br>0,00014940	development delay, motor delay, speech delay, intellectual disability, seizure, microcephaly, failure to thrive, brain atrophy
	c.2635A>T	p.M879L	24.3	n.a.	abdominal distension, intermittent diarrhea
	c.2903A>G	p.E968G	26	69/87187<br>0,00079140	failure to thrive, dysphagia, feeding difficulties, gastroparesis
	c.2969A>G	p.Q990R	24.6	n.a.	developmental regression, global developmental delay, ataxia, seizures, expressive language delay, autism, congenital malrotation of small bowel, dysphagia, vomiting
	c.3028A>G	p.T1010A	26.4	2/80326<br>0,00002490	peripheral neuropathy
	c.3112G>A	p.V1038I	22.4	5/87892<br>0,00005689	delayed motor milestones, delayed speech, developmental regression, intellectual disability, seizure disorder, failure to thrive, feeding difficulties, vomiting

	c.3445C>T	p.Q1149X	36	n.a.	infantile seizures, abnormal brain MRI
	c.3613G>C	p.E1205Q	18.80	22/80262<br>0,00027410	intellectual disability, generalized tonic-clonic seizures, failure to thrive
	c.3736A>G	p.M1246V	24.1	1/7702<br>0,00012984	developmental regression, delayed motor milestones, delayed speech, intellectual disability, sensorineural hearing loss, vision loss, feeding difficulties
	c.3915_3921del	p.D1305Efs*18	35	n.a.	gastroesophageal reflux
	c.4312G>A	p.V1438I	16.56	2/80342<br>0,00002489	delayed motor milestones, delayed speech, developmental regression, failure to thrive, intellectual disability, autistic spectrum disorder, seizure disorder, microcephaly, increased FLAIR sequence in white matter frontal lobes, abnormal EEG
	c.4390A>G	p.I1464V	23.1	133/87937<br>0,00151245	global developmental delay, failure to thrive
	c.4390A>G	p.I1464V	23.1	133/87937<br>0,00151245	global developmental delay, failure to thrive, developmental regression, microcephaly, bilateral sensorineural hearing loss, nystagmus, vision loss, hyperreflexia, febrile seizures, dysphagia, cerebral atrophy
	c.4390A>G	p.I1464V	23.1	133/87937<br>0,00151245	constipation
	c.4424A>G	p.N1475S	17.76	n.a.	migraines, headache, alternating diarrhea, constipation
	c.3389A>T	p.D1130V	32	n.a.	delayed motor milestones, delayed speech, autism, intellectual disability, seizure disorder, GI problems
	c.3346G>C	p.A1116P	32	n.a.	global developmental delay, failure to thrive, microcephaly
	c.3227A>T	p.E1076V	23.60	3/43824<br>0,00006846	failure to thrive, dysphagia, poor suck, malrotation of the intestine, developmental delays,
	c.3034C>T	p.R1012C	27.80	1/77714<br>0,00001287	developmental delay, failure to thrive, infantile spasms, repetitive movements
	c.2972C>T	p.P991L	14.74	126/66208<br>0,00190309	abdominal pain, gastroperesis, chronic constipation, neurological complaints (diffuse weakness, muscle cramps, ANS dysfunction)
	c.2945C>A	p.T982N	24.70	n.a.	global developmental delay, failure to thrive

	c.2782G>A	p.A928T	22.20	0/75278<br>0,00000000	microcephaly, epilepsy, global developmental delay, failure to thrive
	c.2734G>A	p.E912K	33	2/57412<br>0,00003484	profound delayed motor milestones, non-verbal, intractable epilepsy, failure to thrive
	c.2665C>T	p.R889W	25.60	n.a.	Nausea, vomiting, esophagitis, gastroesophageal reflux, gastric ulcer, constipation, migraine
	c.2612C>G	p.P871R	24	1/106652<br>0,00000938	speech delay, hearing loss, failure to thrive
	c.2569T>G	p.F857V	21.90	n.a.	weight loss, progressive motor weakness, proximal to distal weakness accompanied by GI motility, bowel and urinary incontinence, decreased tone of rectum, diverticulosis, sphincter dysfunction
	c.2542G>C	p.D848H	22.90	0/85322<br>0,00000000	learning disability, gastroparesis
	c.2315T>C	p.F772S	32	n.a.	delayed motor milestones, failure to thrive, cerebral hypoplasia and volume loss, delayed myelination, migrational abnormalities
	c.2049del	p.K684Sfs*14	23.30	n.a.	lissencephaly, seizures, failure to thrive, feeding difficulties, poor suck
SREBF1	c.1729G>T	p.G577C	22.40	n.a.	inflammatory bowel disease, migraines
	c.1583A>G	p.N528S	14.72	3/103698<br>0,00002893	meconium aspiration, feeding problems, delayed speech and language development, delayed motor milestones, hypoxic-ischemic encephalopathy, static encephalopathy, congenital spastic diplegia, constipation, bilateral sensorineuronal hearing impairment
	c.1475C>T	p.T492M	20.80	8/113902<br>0,00007024	severe developmental delay, static encephalopathy, epilepsy, feeding issues, microcephaly
	c.1372G>C	p.E458Q	26.20	n.a.	constipation
	c.1244A>G	p.E415G	26.70	0/103850<br>0,00000000	developmental regression, failure to thrive, autism spectrum disorder, speech delay, asthma, GI problems
	c.1205C>T	p.S402L	15.09	9/100196<br>0,00008982	chronic diarrhea with inflammatory bowel disease, failure to thrive
	c.1202T>C	p.V401A	15.96	n.a.	delayed motor milestones, delayed speech, hearing loss,

					intellectual disability, seizure disorder
	c.1163G>A	p.R388H	28.60	n.a.	abdominal pain, emesis, obesity
	c.1112G>C	p.R371P	26.90	n.a.	delayed motor milestones, possible delayed speech, microcephaly, hyperextensibility, failure to thrive
	c.1013C>T	p.S338F	28.70	0/109234<br>0,00000000	abdominal pain, chronic constipation
	c.728A>G	p.H243R	26	n.a.	delayed motor milestones, failure to thrive
	c.607A>G	p.T203A	19.88	1/108680<br>0,00000920	global developmental delay, abnormal deep tendon reflex, nystagmus, failure to thrive
	c.560G>A	p.G187D	20.80	n.a.	delayed motor milestones, diarrhea
	c.298C>A	p.P100T	17.72	n.a.	delayed motor milestones, delayed speech, hearing loss, seizure disorder, microcephaly, failure to thrive, feeding intolerance, structural brain abnormalities
	c.251_253del	p.E84del	15.68	n.a.	lissencephaly, seizures, agenesis of corpus callosum, failure to thrive, hand clenching, feeding difficulties, poor suck
	c.152A>G[#]	p.D51G	15.65	5/109392<br>0,00004571	global developmental delay, intellectual disability, autism, seizure disorder, failure to thrive, gastroesophageal reflux disease, GI dysmotility, ulcerative colitis
	c.64G>C	p.D22H	26	1/77724<br>0,00001287	severe global developmental delay, motor regression, seizures, sensorineural hearing loss, does not walk, microcephaly, failure to thrive, vision loss, non-verbal
ABCD1	c.173C>T	p.S58F	14.06	7/46328<br>0,00015110	delayed motor milestones, delayed speech, intellectual disability, constipation
	c.208G>C	p.V70L	17.02	0/54099<br>0,00000000	motor and speech delay, intellectual disability, inattention, hyperactivity, feeding difficulty, gastroesophageal reflux, constipation, failure to thrive
	c.274G>A	p.G92R	23.2	7/64481<br>0,00010856	failure to thrive, intellectual disability, microcephaly, hearing loss, blindness, small bowel obstruction, intestinal dysmotility, constipation
	c.274G>A	p.G92R	23.2	7/64481<br>0,00010856	motor delay, speech delay, intellectual disability, failure to thrive
	c.274G>A	p.G92R	23.2	7/64481	failure to thrive, autism, ADHD

				0,00010856	
	c.601G>A	p.V201M	17.95	10/87267<br>0,00011459	vomiting, ataxia, balance issues, neuropathic-like foot pain, constipation, migraines, anxiety, developmental delay
	c.601G>A	p.V201M	17.95	10/87267<br>0,00011459	failure to thrive
	c.695C>T	p.A232V	15.14	n.a.	delayed motor milestones, speech problems, progressive cognitive impairment, hearing loss, partial complex seizures, failure to thrive, oromotor feeding disorder, gastroesophageal reflux disorder, abnormal brain MRI
	c.707G>A	p.R236H	25	70/85674<br>0,00081705	global developmental delay, developmental regression, intellectual disability, hearing loss, seizure, abnormal movements, microcephaly, failure to thrive, progressive encephalopathy, vision loss
	c.707G>A	p.R236H	25	70/85674<br>0,00081705	global developmental delay, intellectual disability, seizure disorders, failure to thrive, structural brain abnormalities
	c.756C>A	p.F252L	14.93	0/76345<br>0,00000000	global developmental delay, autism, failure to thrive, seizure like episodes
	c.757C>G	p.L253V	16.31	54/84054<br>0,00064244	delayed motor milestones, delayed speech, intellectual disability, seizure disorder, structural brain abnormalities (cerebellar hypoplasia), ataxia, failure to thrive
	c.757C>G	p.L253V	16.31	54/84054<br>0,00064244	failure to thrive, delayed speech and language development, motor delay, severe intellectual disability, gastroesophageal reflux, constipation
	c.896A>G	p.H299R	25.2	n.a.	global developmental delay, severe seizure disorder, microcephaly, swallowing difficulties, vesicoureteral reflux, volume loss of cerebral white matter and thinning of the corpus callosum
	c.935A>G	p.D312G	18.39	n.a.	bilateral hearing loss, global developmental delay, macrocephaly
	c.949A>T	p.I317F	26.7	n.a.	microcephaly, failure to thrive
	c.1117A>G	p.K373E	13.59	7/80312<br>0,00008716	delayed motor milestones, macrocephaly, failure to thrive
	c.1117A>G	p.K373E	13.59	7/80312	gastroesophageal reflux

				0,00008716	
	c.1172T>C	p.L391P	27.9	n.a.	seizures, nausea, vomiting, diarrhea, constipation
	c.1229C>T	p.T410M	24	2/80310<br>0,00002490	developmental delay, acute mania, intellectual disability, hearing loss, seizures, ataxia, microcephaly, failure to thrive, nystagmus, vision impairment
	c.1354C>T	p.R452W	17.13	48/80219<br>0,00059836	global developmental delay (motor and speech), intellectual disability, seizures, macrocephaly, failure to thrive, structural brain abnormalities , constipation
	c.1354C>T	p.R452W	17.13	48/80219<br>0,00059836	global developmental delay, feeding and swallowing difficulties, failure to thrive, delayed speech
	c.1354C>T	p.R452W	17.13	48/80219<br>0,00059836	microcephaly, constipation, developmental delay
	c.1457G>A	p.G486E	24.2	n.a.	global developmental delay, developmental regression, seizure, intellectual disability, microcephaly, failure to thrive, structural brain abnormalities
	c.1617G>A	p.M539I	23.5	n.a.	delayed motor milestones, no speech, intellectual disability, epilepsy, complex partial seizures, oropharyngeal dysphagia, central vision impairment, thin corpus callosum
	c.1771C>T	p.R591W	24.7	n.a.	headaches, migraines
	c.1794G>T	p.M598I	15.83	1/76307<br>0,00001310	global motor delay, delayed speech, onset of partial complex seizures, bilateral mixed hearing loss, microcephaly, failure to thrive
	c.1810G>A	p.V604I	23.4	19/70488<br>0,00026955	headaches, abdominal pain, GI dysfunction, gastroesophageal reflux disorder, rapid gastric emptying, abnormal findings in the autonomic nerve endings, non-specific white matter gliosis
	c.2035T>A	p.W679R	27.8	n.a.	likely diagnosis of hereditary spastic paraplegia, bladder urgency, stress incontinence
	c.2056T>G	p.S686A	14.39	n.a.	failure to thrive, seizures, developmental regression, diarrhea
	c.2065C>T	p.R689C	29.6	7/69627<br>0,00010054	global developmental delay, intellectual disability, intractable myoclonic absence seizures with strong photosensitivity, failure to thrive

	c.2178_2220del	p.A727Rfs	32	n.a.	Dandy Walker variant, feeding difficulties, microcephaly, agenesis of corpus callosum
	c.2149_2231del	p.C717fs	35	n.a.	delayed motor milestones, intellectual disability
PIAS2	c.1766A>G	p.H589R	20.50	1/109388<br>0,00000914	seizure-like movements, abnormal EEG, feeding difficulties, acid reflux
	c.1685A>G	p.Q562R	26.50	n.a.	nausea, stomach pain
	c.1670T>C	p.I557T	24.40	n.a.	severe intellectual disability, delayed motor milestones, delayed speech, microcephaly, gastroesophageal reflux, hypoplasia of the cerebellum
	c.1699_1701del§	p.S567del	18,29	n.a.	developmental delays, microcephaly, feeding difficulties, failure to thrive
	c.1598_1599insACT§	p.S533delinsYS	22.10	n.a.	intellectual delay with regression, ADD, autism, ataxia, tremors, tics, nausea, constipation, seizures, headaches
	c.1509-4A>G	N/A	22.30	n.a.	failure to thrive, pyloric stenosis
	c.1475T>G	p.F492C	23.10	3/109306<br>0,00002745	failure to gain weight, microcephaly, feeding difficulties, developmental delay
	c.1279A>G§	p.M427V	21.50	2/109370<br>0,00001829	vomiting, dysmotility, seizure disorders
	c.1063A>C	p.I355L	19.85	1/109276<br>0,00000915	feeding difficulties, poor suck, dysphagia
	c.960A>G	p.R320	13.38	n.a.	feeding difficulties
	c.668A>G	p.E223G	24.70	n.a.	developmental delay, motor regression, learning disability, behavioral abnormality, epilepsy, chewing difficulties, weight loss, headache, constipation, white matter volume loss
	c.388T>C	p.F130L	17.75	n.a.	global developmental delay, suspected seizures, microcephaly, visual impairment, nystagmus, bilateral hearing loss, failure to thrive, gastroesophageal reflux disease (GERD), corpus callosum abnormality, deficient myelination
	c.5C>A	p.A2E	24.70	n.a.	delayed motor milestones, delayed speech, autism, intellectual disability, microcephaly, hyperphagia, gastroesophageal reflux disorder
