# Supplementary material for: A complementary study approach unravels novel players in the pathoetiology of Hirschsprung disease
Source: PLoS Genet. 2020 Nov 5;16(11):e1009106. doi: 10.1371/journal.pgen.1009106 (PMC7643938; doi:10.1371/journal.pgen.1009106)
Supplement: S6 Table — Green indicates partial co-expression of both antigens or immunofluorescence signals in close spatial proximity to each other. Red indicates no overlap in respective antigen signals. (PDF) [file pgen.1009106.s008.pdf]

S6 Table: Results of protein expression analyses in murine embryonic tissues

Green indicates partial co-expression of both antigens or immunofluorescence signals in close spatial proximity to each other. Red indicates no overlap in respective antigen signals.

**Stage**	**Marker**	**Cell type**	**Candidate protein**			
			**Atp7a**	**Srebf1**	**Abcd1**	**Pias2**
**E9.5**	Sox10	Pre-enteric NCCs				
**E10.5**	Sox10	ENCDCs				
**E11.5**	Sox10	ENCDCs, immature glia				
	Tubb3	Immature neurons				
	Pgp9.5	Immature neurons				
	HuC/D	Immature neurons				
	ECad	Immature epithelial cells				
**E13.5**	Tubb3	Immature neurons				
	Pgp9.5	Immature neurons				
	HuC/D	Immature neurons				
	Ecad	Immature epithelial cells				
	Sma	Immature muscle cells				
