# Supplementary material for: A complementary study approach unravels novel players in the pathoetiology of Hirschsprung disease
Source: PLoS Genet. 2020 Nov 5;16(11):e1009106. doi: 10.1371/journal.pgen.1009106 (PMC7643938; doi:10.1371/journal.pgen.1009106)
Supplement: S7 Table — (PDF) [file pgen.1009106.s009.pdf]

S7 Table: Summary of validation steps for the four selected candidate genes

GTEx data (https://www.gtexportal.org/home/) were summarized in qualitative manner.

**Type of analysis**	**IPA analysis**	**WES/WGS data of non-HSCR patients**	**GTEx database**	**Immunofluorescence analysis**	**Immunohistochemical analysis**
**Question**	direct and indirect interactions to ENS-relevant and/or HSCR risk factors	association with ENS- and/or CNS-related phenotypes	expression in human brain and colon	expression in murine embryonic ENS-related tissues and cells	expression in human fetal colon and hindgut specimens
ATP7A	yes	yes	yes	yes	yes
SREBF1	yes	yes	yes	yes	yes
ABCD1	yes	yes	yes	yes	yes
PIAS2	yes	yes	yes	yes	yes
