# Supplementary material for: A complementary study approach unravels novel players in the pathoetiology of Hirschsprung disease
Source: PLoS Genet. 2020 Nov 5;16(11):e1009106. doi: 10.1371/journal.pgen.1009106 (PMC7643938; doi:10.1371/journal.pgen.1009106)
Supplement: S8 Table — (PDF) [file pgen.1009106.s010.pdf]

S8 Table: Oligonucleotides

Primer name	Sequence (5' > 3')	Application
ABCD1_Seq_for	AGCAACAATCCTTCCAGCCA	Genotyping missense variant
ABCD1_Seq_rev	GGAATACCCGGTTCATGCCA	
ASCL1_qPCR_for	CGACTTCACCAACTGGTTCTG	qRT PCR
ASCL1_qPCR_rev	ATGCAGGTTGTGCGATCA	
ATP7A_Seq_for	ACACCTTCGGAAGCTGAGTC	Genotyping missense variant
ATP7A_Seq_rev	ACAGGCTTCCAATATGTTAGGTC	
CRISPR_Cas9_rev	GTCTGCAGAATTGGCGCAC	Sequence validation sgRNA cloning
CRISPR_ABCD1_for	TTGAGTTTGAGACCTGGCCC	Genotyping of CRISPR clones
CRISPR_ABCD1_rev	CCACTTGAGCCTGGGAAGTT	
CRISPR_ATP7A_for	TGGTAGAGTGTGAAGTGTTGGAG	Genotyping of CRISPR clones
CRISPR_ATP7A_rev	CCATGAATTGCCAACCCAGC	
CRISPR_PIAS2_Ex2_T1_for	GCTTGAGGGTGACTTGGCTT	Genotyping of CRISPR clones
CRISPR_PIAS2_Ex2_T1_rev	CGGCCAAGTCAGGTTCTACA	
CRISPR_PIAS2_Ex2_T2_for	TGTGGAGGAAATGTTGCCCT	Genotyping of CRISPR clones
CRISPR_PIAS2_Ex2_T2_rev	GGCTAGTGGGAGAGGAGGAG	
CRISPR_PIAS2_Ex2_T3_for	TGATCCTCTTGTGTTGGCCTC	Genotyping of CRISPR clones
CRISPR_PIAS2_Ex2_T3_rev	AGAAACTGGAACCCTTGTGCA	
CRISPR_PIAS2_Ex6_for	TCCCCTCACTTCCCAGTTTG	Genotyping of CRISPR clones
CRISPR_PIAS2_Ex6_rev	TAACTTTAAGAATCTCCACACTGC	
CRISPR_RET_for	AAGCTGTATGTGGACCAGGC	Genotyping of CRISPR clones
CRISPR_RET_rev	CCAGCCTCACTTAACCCCTG	
CRISPR_SREBF1_Ex5_T1_for	TAACGACCACTGTGACCTCG	Genotyping of CRISPR clones
CRISPR_SREBF1_Ex5_T1_rev	ATAGGCAGCTTCTCCGCATC	
GAP43_qPCR_for	CCATGCTGTGCTGTATGAGAA	qRT PCR
GAP43_qPCR_rev	GACAGGGAAAATTCACACTTGAG	
GAPDH_for	CAGCCTCGTCCCGTAGAC	qRT PCR
GAPDH_rev	CGCTCCTGGAAGATGGTG	
GAPDH_for	CGACCACTTTGTCAAGCTCA	

GAPDH_rev	AGGGGTCTACATGGCAACTG	Semiquantitative PCR (reference)
MAP2_qPCR_for	CCTGTGTTAAGCGGAAAACC	qRT PCR
MAP2_qPCR_rev	AGAGACTTTGTCCTTTGCCTGT	
NES_qPCR_for	GAGGTGGCCACGTACAGG	qRT PCR
NES_qPCR_rev	AAGCTGAGGGAAGTCTTGGA	
NPY_qPCR_for	CTCCCCGACAGCATAGTA	qRT PCR
NPY_qPCR_rev	GCCCCAGTCGCTTGTTAC	
P75NTR_qPCR_for	TCATCCCTGTCTATTGCTCCA	qRT PCR
P75NTR_qPCR_rev	TGTTCTGCTTGCAGCTGTTC	
PIAS2_Exon5_for	GCAGAGACAAGTTGCCCTCA	Alternative splicing of edited clone
PIAS2_Exon7_rev	GTGCTCTGGAATGATCAGGGT	
PIAS2_Seq_for	TCCAACACACATTCATCACAGT	Genotyping missense variant
PIAS2_Seq_rev	TCGCAGTGATTGACCATTCCA	
RET_overexpr_cloning_for	gATGGCGAAGGCGACGTC	Cloning overexpression construct
RET_overexpr_cloning_rev	actgtctagaTTAACTATCAAACGTGTCCAT TAATTTTGC	
RET_rs2506030_rev	AGAGCAAAACGACCCATGAGT	
SDHA_qPCR_for	TGGGAACAAGAGGGCATCTG	qRT PCR
SDHA_qPCR_rev	CCACCACTGCATCAAATTCATG	
SEMA_rs11766001_rev	TGCCTCCTTTGTAGCTTCTTGT	
SREBF1_FL_overexpr_ cloning_for	actgggatccATGGACGAGCCACCCTTCA	Cloning overexpression construct
SREBF1_FL_overexpr_ cloning_rev	actgtctagaTAGCTGGAAGTGACAGTGGT	
SREBF1_A_SEQ_for	CAGCCTCCCTTGGTATCACA	Genotyping compound heterozygous missense variants
SREBF1_A_SEQ_rev	GTCAGGCAGTGGTGGAGATG	
SREBF1_B_SEQ_for	CACAGGGTTGAGGCCAGAG	
SREBF1_B_SEQ_rev	CTTCCCAGTGGCCCTAGGT	
SYP_qPCR_for	CCAATCAGATGTAGTCTGGTCAGT	qRT PCR
SYP_qPCR_rev	AGGGGTGGAGACCTAGGGTA	
TAU_qPCR_for	GGACTGGAAGCGATGACAA	qRT PCR
TAU_qPCR_rev	GGCTAAGGCAAGGCCTATTT	

TUBB3_qPCR_for	GCAACTACGTGGGCGACT	qRT PCR
TUBB3_qPCR_rev	ATGGCTCGAGGCACGTACT	
UCHL1_qPCR_for	AGATCAACCCCGAGATGCT	qRT PCR
UCHL1_qPCR_rev	ACCGAGCCCAGAGACTCC	
