# Supplementary material for: A complementary study approach unravels novel players in the pathoetiology of Hirschsprung disease
Source: PLoS Genet. 2020 Nov 5;16(11):e1009106. doi: 10.1371/journal.pgen.1009106 (PMC7643938; doi:10.1371/journal.pgen.1009106)
Supplement: S9 Table — (PDF) [file pgen.1009106.s011.pdf]

S9 Table: Primary antibodies

Antibody	Subtype	Supplier	Annotations (Dilution, permeabilization, demasking)			
			Cells	Cryosections tissue	FFPE tissue sections	Western blot
**Rabbit anti-ABCD1**	Mouse, rat, human	Abcam, ab197013	-	1:100 (0.1 % Tween)	1:100	1:5000
**Rabbit anti-ATP7A**	Mouse, rat, rabbit, human	Abcam, ab125137	-	1:75 (0.1% Triton)	-	1:250
**Rabbit anti-ATP7A**	Mouse, rat, human	Elabscience, E-AB-16268	-	-	1:75	-
**Mouse anti-ECad**	Mouse, rat, horse, human	Abcam, ab76055	-	1:100 (0.1% Triton, demasking)	-	-
**Mouse anti-GAPDH**	Mouse, rat, human, chicken, xenopus, dog, fish, pig,	Abcam, ab8245	-	-	-	1:2000
**Rabbit anti-GAP43**	Mouse, rat, human	Abcam, ab75810	1:100	-	-	-
**Mouse anti-HuC/D-Biotin**	Mouse, human	Molecular Probes, A-21272	-	1:100 (0.1% Triton)	-	-
**Rabbit anti-MAP2**	Mouse, rat, human, monkey	Cell signalling, 4542	1:35	-	-	-
**Mouse anti-PGP9.5**	Mouse, rat, human, rabbit, sheep, zebrafish	Abcam, ab8189	-	1:100 (0.1% Triton)	-	-
**Rabbit anti-PIAS2**	Mouse, rat, human, chicken	Biozol, GTX115180	-	1:100 (0.1% Triton, demasking)	1:500	-
**Rabbit anti-RET**	Mouse, rat, human	Abcam, ab134100	-	-	-	1:1000
**Mouse anti-SMA**	Mouse, rat, human, Bovine, chicken, rabbit, baboon	Abcam, ab18147	-	1:250 (0.1% Triton)	-	-
**Mouse anti-SOX10**	Mouse, rat, human	Affymetrix, 14-5923	-	1:50 (0.1% Triton)	-	-
**Rabbit anti-SREBF1**	Mouse, rat, human	Abcam, ab28481	-	-	1:1000	-
**Rabbit anti-SREBF1**	Mouse, rat, human, pig, hamster	Abnova, PAB12021	-	1:300 (1% Triton)	-	1:500

**Rabbit anti-SYP**	Mouse, rat, human	Abcam, ab32127	1:100	-	-	-
**Mouse anti-TAU**	Mouse, rat, human, predicted: cow	Cell signalling, 4019	1:150	-	-	-
**Mouse anti-TUBB3**	mammals	Promega, G7121	1:500	1:500<br>(0.1% Triton)	-	-
