# Supplementary material for: A complementary study approach unravels novel players in the pathoetiology of Hirschsprung disease
Source: PLoS Genet. 2020 Nov 5;16(11):e1009106. doi: 10.1371/journal.pgen.1009106 (PMC7643938; doi:10.1371/journal.pgen.1009106)
Supplement: S10 Table — (PDF) [file pgen.1009106.s012.pdf]

S10 Table: Secondary antibodies

Antibody	Conjugate	Supplier	Annotations (Dilution)	
			Immuno-fluorescence	Western blot
**Goat anti-mouse IgG**	Alexa Fluor 488	Thermo Fisher Scientific A-11029	1:250	-
**Goat anti-mouse IgG**	Alexa Fluor 647	Thermo Fisher Scientific A-21236		
**Goat anti-rabbit IgG**	Alexa Fluor 568	Thermo Fisher Scientific A-11011		
**Goat anti-rabbit IgG**	Peroxidase	Jackson ImmunoResearch 111-035-144	1:200	
**Streptavidin**	Alexa Fluor 488	Thermo Fisher Scientific S32354	1:250	
**Donkey anti-rabbit**	IRDyeCW680	LI-COR 926-32223		1:10.000
**Donkey anti-mouse**	IRDyeCW800	LI-COR 926-32212		
