# Supplementary material for: A complementary study approach unravels novel players in the pathoetiology of Hirschsprung disease
Source: PLoS Genet. 2020 Nov 5;16(11):e1009106. doi: 10.1371/journal.pgen.1009106 (PMC7643938; doi:10.1371/journal.pgen.1009106)
Supplement: S11 Table — (PDF) [file pgen.1009106.s013.pdf]

S11 Table: sgRNA

Gene	Exon (Ex)	Sequence (5'>3') (Overhang nucleotides necessary for cloning into the vector backbone are lower-cased)
RET	Ex2	caccgTCTACGGCACGTACCGCACA
		aaacTGTGCGGTACGTGCCGTAGAc
ATP7A	Ex3	caccGATCATAGTATGGCTCAAGC
		aaacGCTTGAGCCATACTATGATC
SREBF1	Ex5	caccGAGACCTGCCGCCTTCACAG
		aaacCTGTGAAGGCGGCAGGTCTC
ABCD1	Ex2	caccgCGCTCCACACATACTTCATG
		aaacCATGAAGTATGTGTGGAGCGc
PIAS2	Ex6	caccgTAATATTCAAGGGGCGTCCA<br>aaacTGGACGCCCCTTGAATATTAc
	Ex2_T1	caccgCGATATCCACGAACTCTTGA<br>aaacTCAAGAGTTCGTGGATATCGc
	Ex2_T2	caccgATCGGCGTCTATACAATTCT<br>aaacAGAATTGTATAGACGCCGATc
	Ex2_T3	caccGAAAGTCCTTCAAGAGTTCG<br>aaacCGAACTCTTGAAGGACTTTC
