# Supplementary material for: A complementary study approach unravels novel players in the pathoetiology of Hirschsprung disease
Source: PLoS Genet. 2020 Nov 5;16(11):e1009106. doi: 10.1371/journal.pgen.1009106 (PMC7643938; doi:10.1371/journal.pgen.1009106)
Supplement: S12 Table — (PDF) [file pgen.1009106.s014.pdf]

S12 Table: Oligonucleotides used for off-target analysis

Primer name	Sequence (5‘>3‘)	Application
ARHGAP24_for	TGACGGAAGGTTAAAGGCACA	
ARHGAP24_rev	TTCCCCTGCTTCACTGTTGA	
DYRK1A_for	GCTTTTGTGGGTGTAAGGGC	
DYRK1A_rev	CCCAGGAGTCAGTTTGCAGT	
EGF_for	GATGACACTTGGGAGCCTGG	
EGF_rev	GGCTGGTCTCAAACTCCTGG	
NDRG3_for	GTCAGGCCAAGTGTACCCTC	Off-targets_CRISPR *ATP7A*
NDRG3_rev	GGCCTCAGTTACCCCATCTG	
RP11-146N_for	TGCCACGATTCTTCCTCTGG	
RP11-146N_rev	ACTGACCTACTGCCCTGCTA	
SMOX_for	GTCCTGTCTTCCCCATGCAG	
SMOX_rev	TATATGCCCCTTGGTGGTGC	
ZMAT5_for	CAACAACGCTGACTGAAGGC	
ZMAT5_rev	CCTTCCTTCTGCAACTGGGT	
ST8SIA5_rev	TCCTCCTCCCAGCACTAACA	
ST8SIA5_for	TTAACAGCCAAGCCACACCA	
CAMK1G_for	TCAGTAAACACGGACCGAGC	
CAMK1G_rev	TGGCATTTCTGAGCCTCCTG	
CLIC6_2_for	TTAGGTGTTTGGGTGTCGGG	
CLIC6_2_rev	CCATTGAGTCCTCCCGCAAA	
CTDSP1_for	TGTAGAGACAGAGGGCAGCT	Off-targets_CRISPR *SREBF1*
CTDSP1_rev	CGCTTTCCTCTCCCTCTGTG	
EEF1A2_2_for	GTTGAGGTTCAGGTCTGGGG	
EEF1A2_rev	CCGCTCTTCTTCTCCACGTT	
MIR8073_for	ATCCTGGGCTGCCTCTATCA	
MIR8073_rev	AAGCCTGGAACCTACACAGC	
XYLB_for	GAGCCCTGTGGTTTGCTTTG	
XYLB_rev	AACCGGGTTGCAGAAGATGT	
SNORA23_for	TGTATTCCCTCTCTTCAGCAGTG	Off-targets_CRISPR *ABCD1*
SNORA23_rev	ACCCAGCAAGCCTTTTGTAAAA	
