# Supplementary material for: A complementary study approach unravels novel players in the pathoetiology of Hirschsprung disease
Source: PLoS Genet. 2020 Nov 5;16(11):e1009106. doi: 10.1371/journal.pgen.1009106 (PMC7643938; doi:10.1371/journal.pgen.1009106)
Supplement: S1 Fig — Flowchart showing the WES analysis approach and the subsequent steps involved in candidate gene A) identification and selection, B) prioritization as well as C) validation. Technical details and cut offs are given. MAF: Minor allele frequency, comp.: compound. (PDF) [file pgen.1009106.s016.pdf]

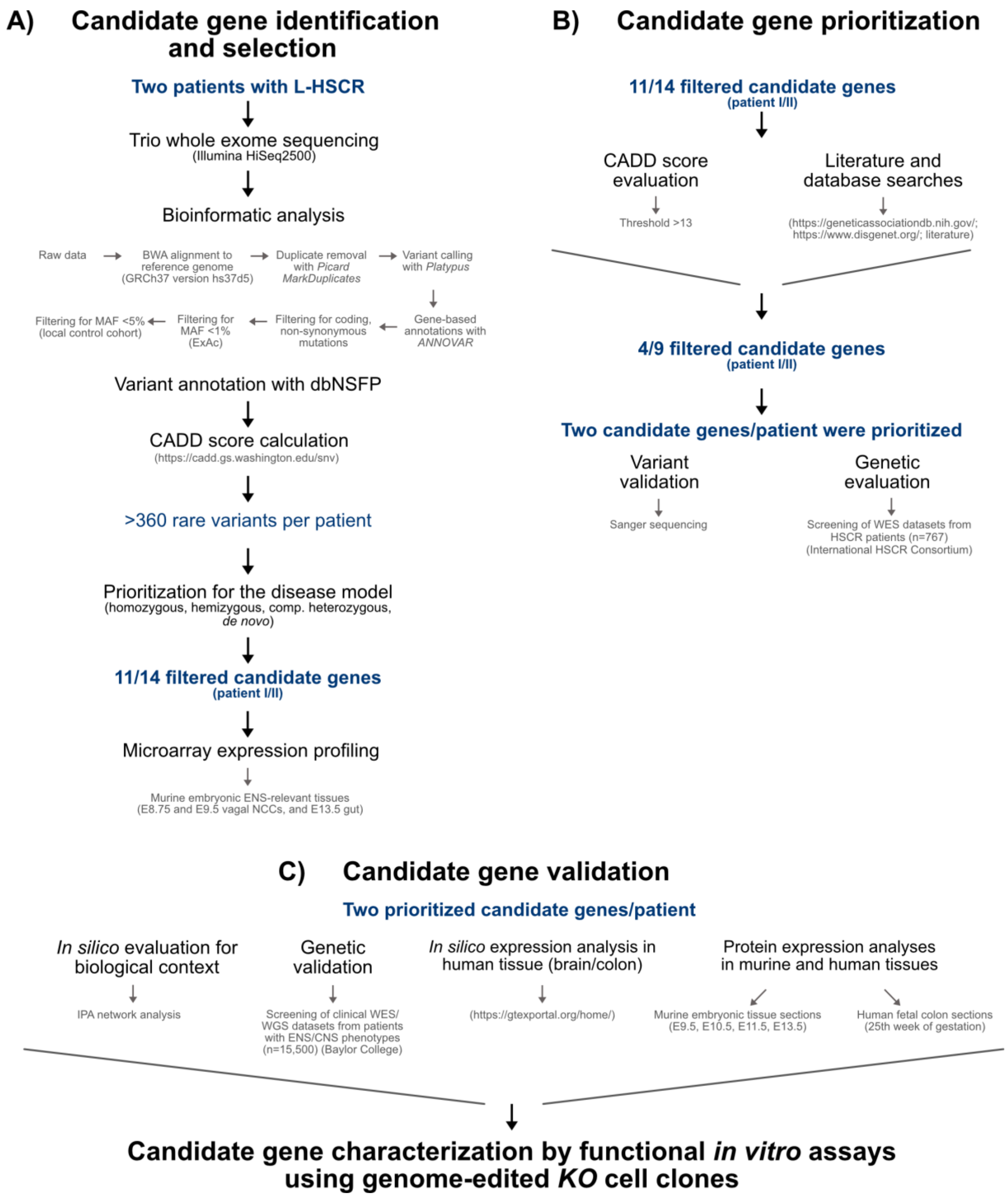

S1 Fig: Components of our comparative study pipeline.

Flowchart showing the WES analysis approach and the subsequent steps involved in candidate gene A) identification and selection, B) prioritization as well as C) validation. Technical details and cut offs are given. MAF: Minor allele frequency, comp.: compound.
