# Supplementary material for: A complementary study approach unravels novel players in the pathoetiology of Hirschsprung disease
Source: PLoS Genet. 2020 Nov 5;16(11):e1009106. doi: 10.1371/journal.pgen.1009106 (PMC7643938; doi:10.1371/journal.pgen.1009106)
Supplement: S2 Fig — Four colored chromatograms are shown for all members of family I (A) and family II (B). In case of heterozygous states, ambiguity codes are given. (PDF) [file pgen.1009106.s017.pdf]

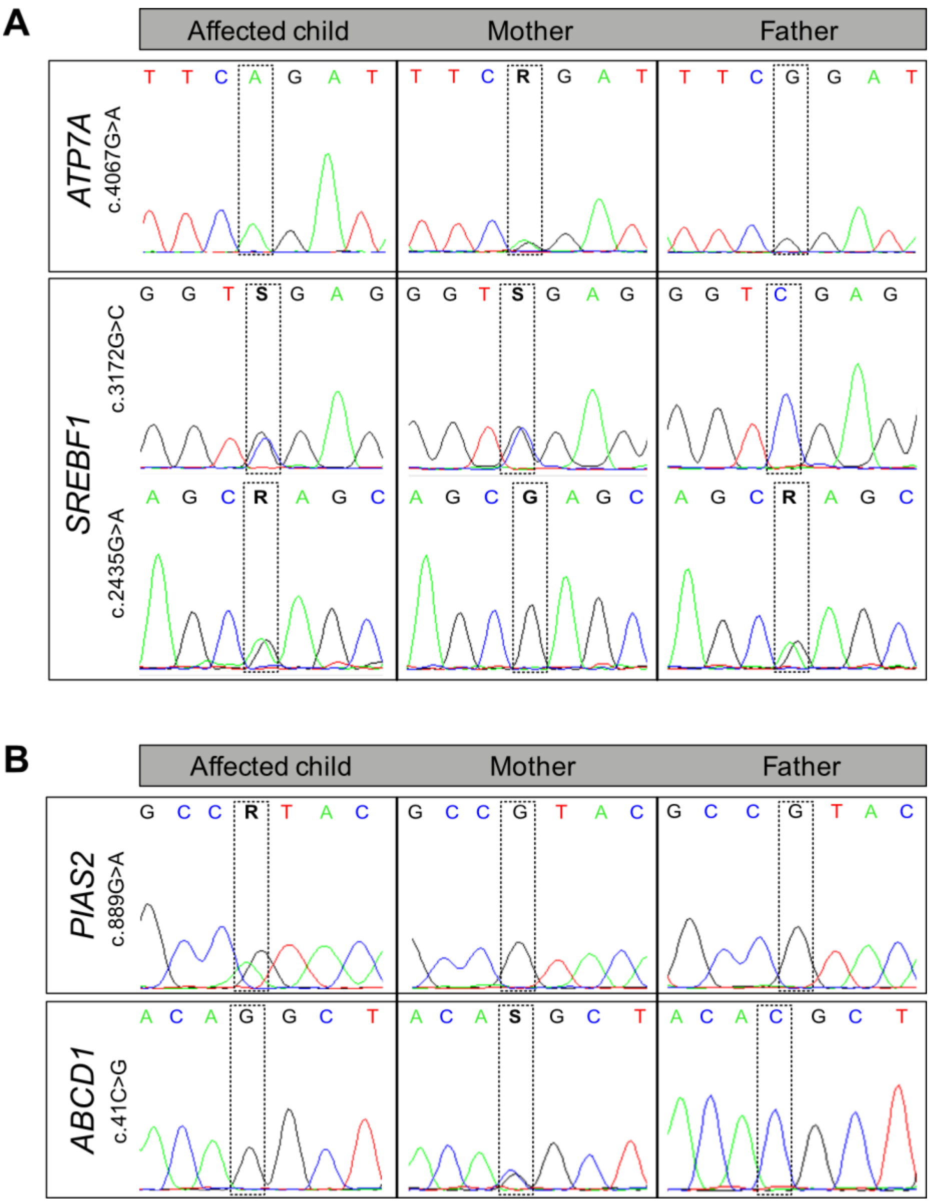

S2 Fig: Variant validation in selected candidate genes by Sanger sequencing.

Four colored chromatograms are shown for all members of family I (A) and family II (B). In case of heterozygous states, ambiguity codes are given.
