# Supplementary material for: A complementary study approach unravels novel players in the pathoetiology of Hirschsprung disease
Source: PLoS Genet. 2020 Nov 5;16(11):e1009106. doi: 10.1371/journal.pgen.1009106 (PMC7643938; doi:10.1371/journal.pgen.1009106)
Supplement: S3 Fig — (A) Expression analyses of each candidate with the NCC marker Sox10 (green) in murine embryos of stage E9.5 are shown. Sox10+ cells in close proximity to the neck are displayed. Cranial and caudal positions are indicated (Atp7a (A'), Srebf1 (A"), Abcd1 (A"'), Pias2 (A""), all in red). Immunofluorescence signals in close spatial proximity suggesting co-expression, are indicated by white arrowheads. (B) Triple staining in the developing midgut at E13.5 reveals expression of all candidates within or in close spatial proximity to immature neurons (Tubb3 (green)) and immature smooth muscle cells (Sma (cyan)); (Atp7a (B'), Srebf1 (B"), Abcd1 (B"'), Pias2 (B""), all in red) (white arrowheads). The gut lumen is highlighted. (A, B) Bottom images show magnifications of the areas highlighted in the top images. Nuclei were counterstained with Hoechst 33342 (blue). Scale bars: 50 μm. (C) Immunohistochemistry analyses in human fetal colon sections show candidate protein expression in enteric ganglia (marked by a red arrowhead) except for ABCD1 (C"'). Bottom images illustrate the gut epithelial layer where candidates are expressed in enterocytes (ATP7A (C'), SREBF1 (C"), PIAS2 (C""), all in brown). Nuclei were counterstained with hematoxylin (blue). Scale bars: 20 μm. (PDF) [file pgen.1009106.s018.pdf]

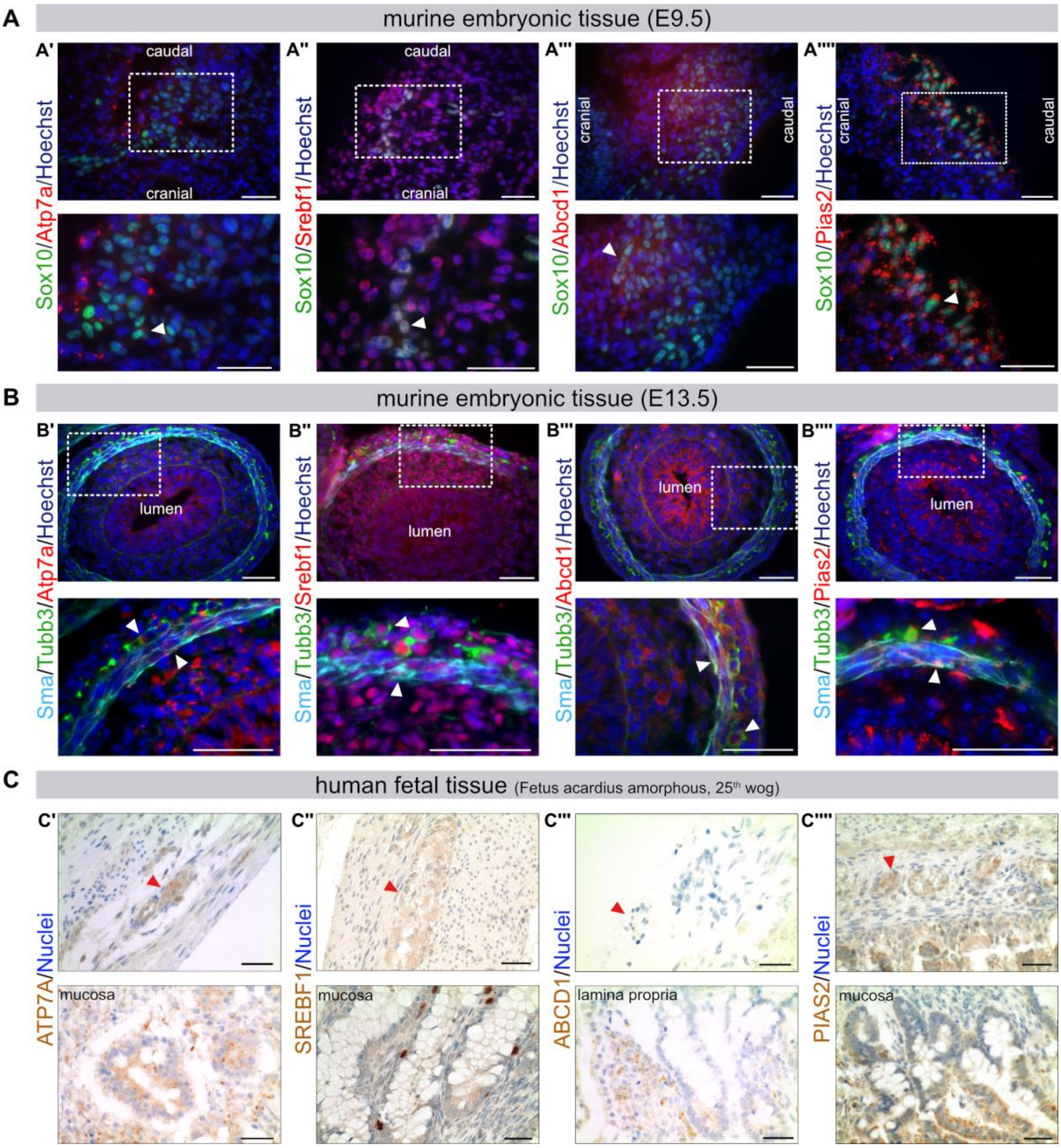


**S3 Fig: Protein expression analyses of candidates in murine embryonic and human fetal tissue sections.**

(A) Expression analyses of each candidate with the NCC marker Sox10 (green) in murine embryos of stage E9.5 are shown. Sox10+ cells in close proximity to the neck are displayed. Cranial and caudal positions are indicated (Atp7a (A'), Srebf1 (A''), Abcd1 (A'''), Pias2 (A''''), all in red). Immunofluorescence signals in close spatial proximity suggesting co-expression, are indicated by white arrowheads. (B) Triple staining in the developing midgut at E13.5 reveals

expression of all candidates within or in close spatial proximity to immature neurons (Tubb3 (green)) and immature smooth muscle cells (Sma (cyan)); (Atp7a (B’), Srebf1 (B’’), Abcd1 (B’’’), Pias2 (B’’’’), all in red) (white arrowheads). The gut lumen is highlighted. (A, B) Bottom images show magnifications of the areas highlighted in the top images. Nuclei were counterstained with Hoechst 33342 (blue). Scale bars: 50 µm. (C) Immunohistochemistry analyses in human fetal colon sections show candidate protein expression in enteric ganglia (marked by a red arrowhead) except for ABCD1 (C’’’). Bottom images illustrate the gut epithelial layer where candidates are expressed in enterocytes (ATP7A (C’), SREBF1 (C’’), PIAS2 (C’’’’), all in brown). Nuclei were counterstained with hematoxylin (blue). Scale bars: 20 µm.
