# Supplementary material for: A complementary study approach unravels novel players in the pathoetiology of Hirschsprung disease
Source: PLoS Genet. 2020 Nov 5;16(11):e1009106. doi: 10.1371/journal.pgen.1009106 (PMC7643938; doi:10.1371/journal.pgen.1009106)
Supplement: S4 Fig — Published RNA sequencing data [2] were evaluated for the expression of selected candidates. Bar chart shows reads per kilobase of exon model per million mapped reads (RPKM) in human fetal hindgut specimens of embryonic week (EW) 12, 14, and 16. (PDF) [file pgen.1009106.s019.pdf]

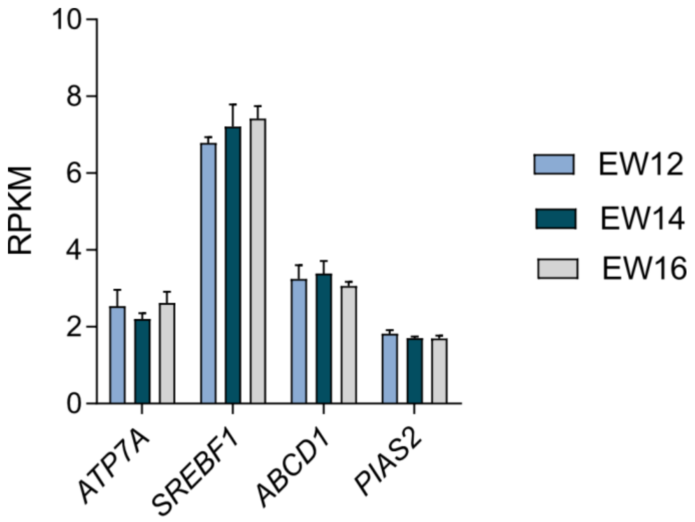

S4 Fig: RNA expression of selected candidates in human fetal colon tissue.

Published RNA sequencing data [1] were evaluated for the expression of selected candidates. Bar chart shows reads per kilobase of exon model per million mapped reads (RPKM) in human fetal hindgut specimens of embryonic week (EW) 12, 14, and 16.

References

1. McCann CJ, Alves MM, Brosens E, Natarajan D, Perin S, Chapman C, et al. Neuronal Development and Onset of Electrical Activity in the Human Enteric Nervous System. Gastroenterology. 2019;156(5):1483-95.e6.
