# Supplementary material for: A complementary study approach unravels novel players in the pathoetiology of Hirschsprung disease
Source: PLoS Genet. 2020 Nov 5;16(11):e1009106. doi: 10.1371/journal.pgen.1009106 (PMC7643938; doi:10.1371/journal.pgen.1009106)
Supplement: S5 Fig — (A'–D') Gene-specific sgRNAs were designed against marked exons. Cas9-mediated double-strand breaks were repaired by NHEJ causing homozygous or compound heterozygous genome modifications at the respective positions, as verified by Sanger sequencing. Four color chromatograms are shown for the genes (RET (A'), ATP7A (B'), SREBF1 (C'), ABCD1 (D')). Genome editing for PIAS2 did not work. PAM sites are underscored. Putative Cas9-cutting sites are marked by red arrowheads. (A"–D") Knockout (KO) on protein level was validated by Western blot analyses using different protein lysates as internal controls (HEK293TN cells transiently transfected with a gene-specific, tagged overexpression construct, SHSY5Y cells, mock control cells) (RET (A"), ATP7A (B"), SREBF1 (C"), ABCD1 (D")). GAPDH was used as a loading control. Predicted protein sizes are annotated. Images show modified blots as individual lanes of respective blots were rearranged if necessary. FL: full length, MAT: mature. (PDF) [file pgen.1009106.s020.pdf]

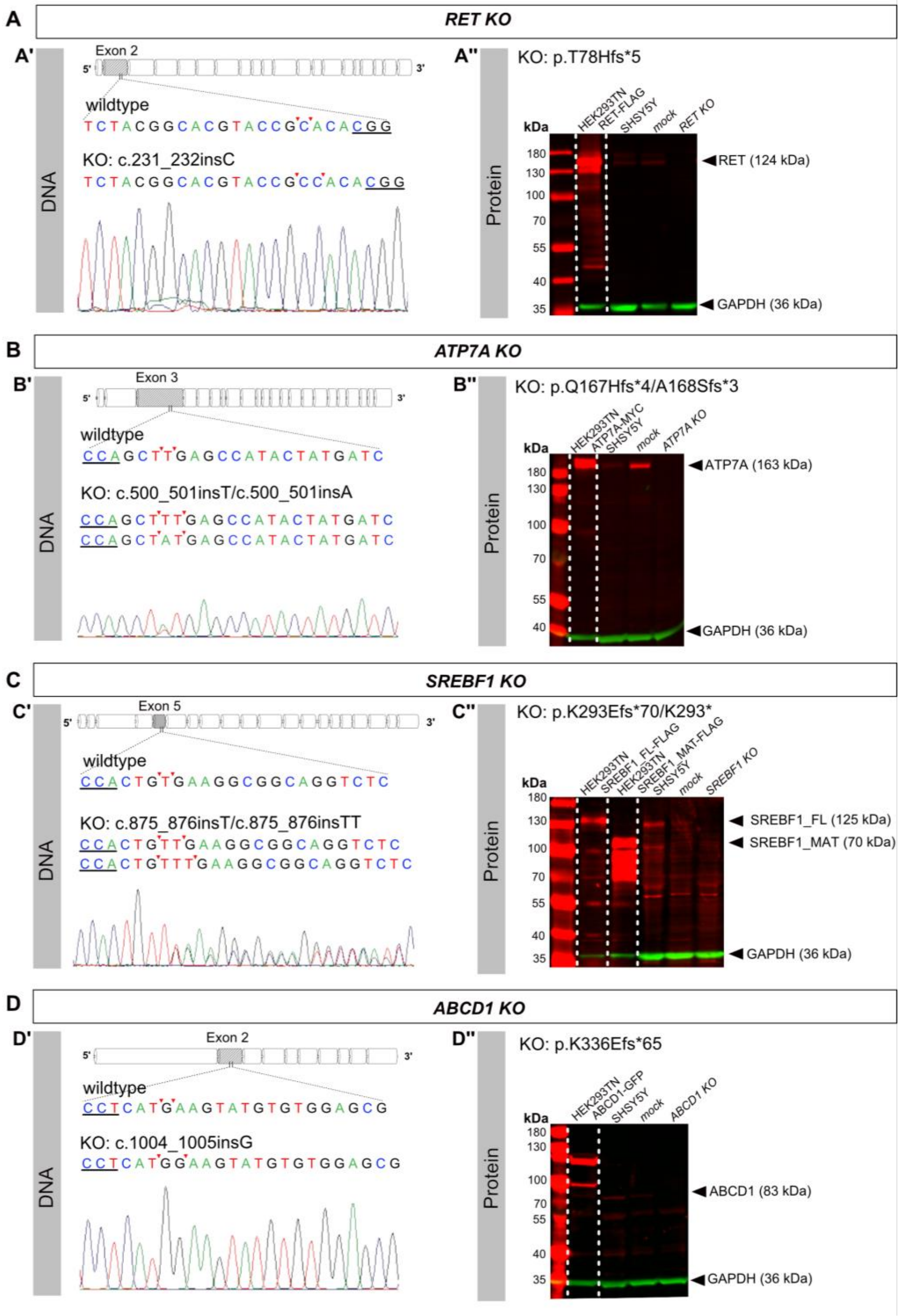


**S5 Fig: CRISPR/Cas9 gene mediated *KO* of *RET* and candidate genes.**

(A'–D') Gene-specific sgRNAs were designed against marked exons. Cas9-mediated double-strand breaks were repaired by NHEJ causing homozygous or compound heterozygous genome modifications at the respective positions, as verified by Sanger sequencing. Four color

chromatograms are shown for the genes (*RET* (A'), *ATP7A* (B'), *SREBF1* (C'), *ABCD1* (D')). Genome editing for *PIAS2* did not work. PAM sites are underscored. Putative Cas9-cutting sites are marked by red arrowheads. (A''–D'') *Knockout (KO)* on protein level was validated by Western blot analyses using different protein lysates as internal controls (HEK293TN cells transiently transfected with a gene-specific, tagged overexpression construct, SHSY5Y cells, *mock control* cells) (RET (A''), ATP7A (B''), SREBF1 (C''), ABCD1 (D'')). GAPDH was used as a loading control. Predicted protein sizes are annotated. Images show modified blots as individual lanes of respective blots were rearranged if necessary. FL: full length, MAT: mature.
