# Supplementary material for: A complementary study approach unravels novel players in the pathoetiology of Hirschsprung disease
Source: PLoS Genet. 2020 Nov 5;16(11):e1009106. doi: 10.1371/journal.pgen.1009106 (PMC7643938; doi:10.1371/journal.pgen.1009106)
Supplement: S6 Fig — Gene expression profiles of different cell fate markers such as P75NTR and NES (neuronal progenitor markers) as well as UCHL1 and ASCL1 (advanced neuronal cell fate markers) in differentiating genome-edited cell clones by qRT PCR analysis. (n = 3, mean + standard error of mean; exploratory data analysis by two-sided unpaired t-Test with/without Welch’s correction, *p<0.05; **p<0.01; ***p<0.001). (PDF) [file pgen.1009106.s021.pdf]

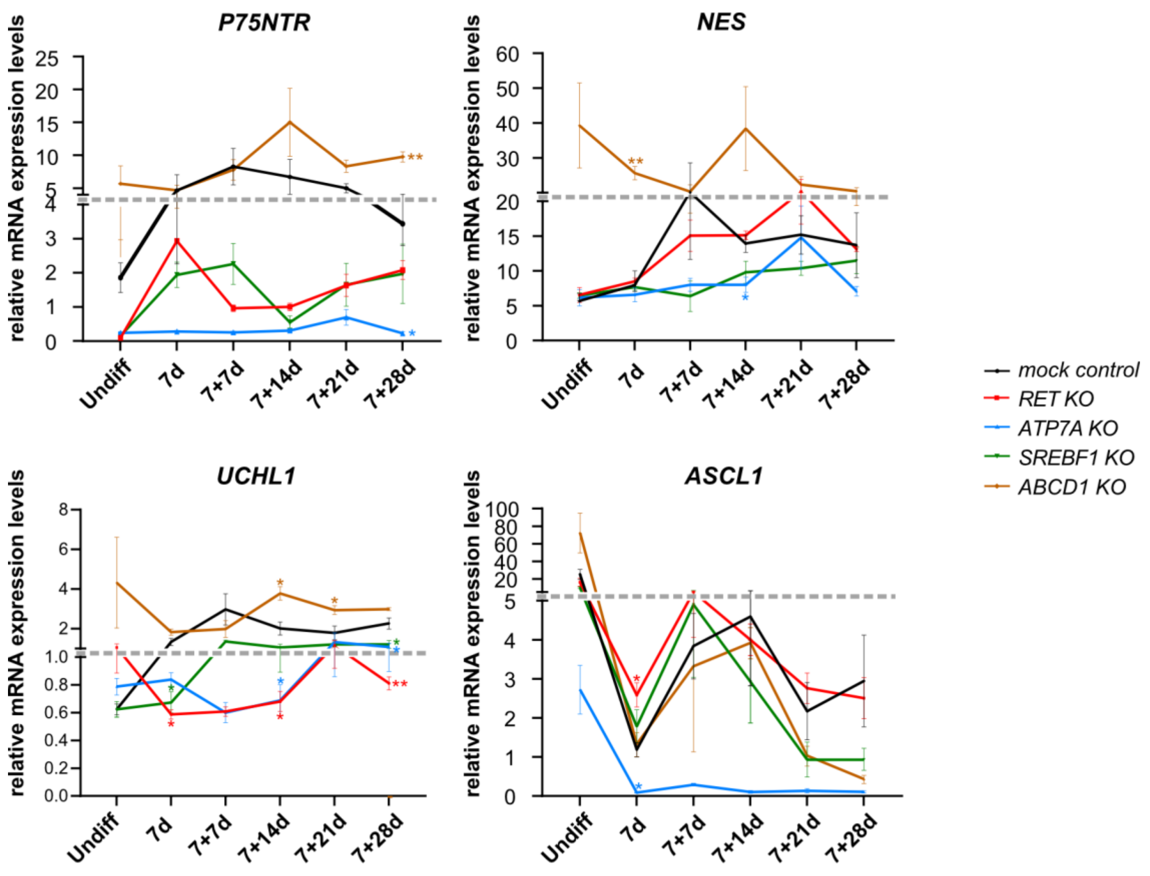

S6 Fig: qRT PCR expression profiling of selected cell markers in differentiating cell clones.

Gene expression profiles of different cell fate markers such as *P75NTR* and *NES* (neuronal progenitor markers) as well as *UCHL1* and *ASCL1* (advanced neuronal cell fate markers) in differentiating genome-edited cell clones by qRT PCR analysis. (n=3, mean + standard error of mean; exploratory data analysis by two-sided unpaired t-Test with/without Welch's correction, *p<0.05; **p<0.01; ***p<0.001).
